# Kinetically Arrested SERS‐Active Aggregates for Biosensing

**DOI:** 10.1002/chem.202500915

**Published:** 2025-06-17

**Authors:** Natalie S. Potter, Kamil Sokołowski, Renata Lang Sala, Jade A. McCune, Oren A. Scherman

**Affiliations:** ^1^ Melville Laboratory for Polymer Synthesis Yusuf Hamied Department of Chemistry University of Cambridge Lensfield Road Cambridge CB2 1EW UK

**Keywords:** biosensors, disassembly, kinetically arrested aggregates, self‐assembly, surface‐enhanced Raman spectroscopy (SERS)

## Abstract

There is great demand for biosensors capable of long‐term, non‐invasive monitoring of low analyte concentrations in various biofluids for diagnostics, personalized healthcare, and disease monitoring. However, biofluids present several challenges to biosensor sensing and stability on account of their complex composition and dynamic nature. Surface‐enhanced Raman spectroscopy (SERS) offers ultra‐high sensitivity, non‐destructive, real‐time monitoring, and non‐invasive detection, ideal for sensing in complex media. Plasmonic gold nanoparticle (AuNP) SERS‐active assemblies have been widely explored owning to their biocompatibility and tunable sensitivity based on AuNP morphology. Here, we employ thiolated poly(ethylene glycol) (PEG‐SH) to produce kinetically trapped metastable AuNP:cucurbit[7]uril (CB[7]) aggregates containing robust nanogaps (ca. 1 nm), which have previously displayed strong, reproducible SERS signals at low concentrations. The AuNP:CB[7]:PEG aggregates maintain strong SERS‐activity, demonstrating low concentration detection of a model Raman reporter and a bioanalyte in a range of biofluids. Moreover, the aggregates are highly tunable through facile modification of the PEG‐SH chain length and grafting density and possess an encoded disassembly mechanism within their design highlighting the potential application of these aggregates for non‐invasive in vivo long‐term monitoring alongside future customized SERS biosensing applications.

## Graphical abstract

Three chemically benign components (AuNP, CB[7] and PEG‐SH) are employed to produce highly‐stable SERS‐active AuNP aggregates able to detect (bio)analytes at low concentrations in high salinity and protein‐containing media. Tunable disassembly is embedded in the AuNP aggregate design, which can be triggered by endogenous thiol‐containing compounds.

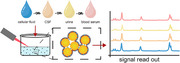

## 1 Introduction

Self‐assembly of plasmonic nanoparticles (NPs) to yield functional aggregates has been widely explored for a range of applications, including nanophotonics, optoelectronics, photocatalysis, as well as surface‐enhanced Raman spectroscopy (SERS), (bio)sensing, drug delivery, and (nano)theranostics.^[^ ^1^, ^2^, ^3^ ^]^ To date, methods to trigger NP assembly have relied on the aggregation of constituent particles by attractive van der Waals forces,^[^ ^4^, ^5^ ^]^ multitopic ligands,^[^ ^6^ ^]^ or biological moieties such as biotin–streptavidin^[^ ^7^ ^]^ and DNA strands.^[^ ^8^, ^9^ ^]^ Although these assembly processes can be rationally designed, once induced they often continue assembling until the nanoparticulate components are consumed yielding colloidal crystals,^[^ ^3^, ^4^, ^6^ ^]^ complex solids,^[^ ^10^ ^]^ or amorphous precipitates^[^ ^10^ ^]^ as terminal thermodynamic products. As a result, achieving colloidal stabilization of NP aggregates prior to reaching the thermodynamic product (i.e., in their metastable state) remains challenging and largely underreported for aqueous systems.^[^ ^11^, ^12^, ^13^, ^14^, ^15^ ^]^

An attractive pathway to achieve colloidal stability while maintaining metastable character of resultant aggregates is through kinetic arrest.^[^ ^16^ ^]^ Recently, we reported the first example of using kinetic arrest to yield metastable plasmonic gold nanoparticle (AuNP) assemblies within photoactive quantum dot (QD) arrays through interfacial self‐limiting aggregation.^[^ 1^4^ ^]^ The initial self‐assembly of AuNPs was triggered by the addition of macrocyclic cucurbit[*n*]uril (CB[*n*]; *n* = 5–8) moieties, which can act as a “molecular glue” to yield robust AuNP:CB[*n*] assemblies with uniform spacing.^[^ ^17^, ^18^, ^19^, ^20^, ^21^ ^]^ QDs acted as surfactants, modulating the AuNP:CB[*n*] assemblies by passivating interfaces and stabilizing kinetically arrested states.^[^ ^14^ ^]^ The resulting hybrid AuNP:CB[*n*]:QD assemblies were permeable and able to efficiently sequester small molecules within their interstitial spaces, allowing real‐time SERS monitoring of chemical reactions.

We postulated that a similar approach could be employed to achieve SERS‐active AuNP aggregates for biosensing applications. Sensing in biological fluids presents a significant challenge on account of their complex and variable composition, often characterized by high salinity and the presence of large components such as proteins.^[^ ^22^ ^]^ High saline environments can trigger irreversible aggregation of metallic nanoparticles (NPs), leading to eventual coalescence and dramatically reducing SERS sensitivity.^[^ ^23^ ^]^ Although non‐specific protein binding can passivate the surfaces of the NPs to stabilize the aggregates, it can also reduce the permeability of the aggregates hindering surface availability and analyte access to the hot‐spots.^[^ ^24^ ^]^ This type of surface coverage ultimately inhibits direct biomarker detection limiting SERS sensors to pre‐labeled systems.

For monomeric metallic nanoparticles, these obstacles are often minimized through the use of ligands decorating the surface. Poly(ethylene glycol) (PEG) ligands are one of the most commonly utilized polymers for NP surface functionalization. Previous reports have shown that an attachment of thiolated PEG to monomeric AuNPs improves overall stability and circulation time of NPs in vivo.^[^ ^25^, ^26^ ^]^ This increase in stability and circulation time is a result of steric interference to prevent non‐specific protein binding as well as enhancing stability in salt environments.^[^ ^25^, ^27^, ^28^ ^]^

Facile clearance of NP aggregates is another critical factor to consider in the design of SERS‐active substrates for in vivo applications.^[^ ^29^, ^30^ ^]^ It has been generally reported that while small NPs (with diameters <10 nm) are rapidly cleared through the kidneys, larger NPs (e.g. >200 nm) risk triggering an inflammatory response causing uptake and clearance of the NPs by macrophages.^[^ ^31^, ^32^, ^33^, ^34^, ^35^, ^36^ ^]^ Particles between 10 and 100 nm have been reported to accumulate in the liver or spleen and are then cleared either by mononuclear phagocytes or processed by the liver and excreted.^[^ ^34^, ^37^ ^]^ To achieve optimal SERS enhancement, previous studies have suggested that optimal size of NPs ranges from 30–100 nm.^[^ ^38^, ^39^ ^]^ Therefore, to design an aggregated system with high SERS enhancement the utilization of larger NPs is often necessary. However, a disassembly mechanism embedded within the design is also essential to allow for potential clearance. Current methods for disassembly often rely on an external trigger such as light or a change in the pH environment.^[^ ^40^, ^41^, ^42^, ^43^ ^]^ However, these external triggers either involve aggregates that show no significant LSPR absorbance in the near‐IR range (e.g. 650–900 nm), which is essential for deep tissue SERS sensing in the biological regime, or the pHs employed are outside the physiological range.

Herein, we confront these challenges and produce SERS‐active kinetically arrested AuNP:CB[*n*] aggregates comprised of biologically‐benign components. Thiol‐terminated poly(ethylene glycol) (PEG‐SH) ligands were added to emerging AuNP:CB[*n*] aggregates, inducing kinetic arrest. By fine‐tuning the ratio of the chemical components, a wide stability window was accessed (hours to weeks) for extended analyte sensing and variable disassembly kinetics (minutes to days). To address the difficulties working in biological media, stability of the AuNP:CB[*n*]:PEG aggregates in biofluids with high salinity and in the presence of proteins was investigated. The SERS activity of these tunable aggregates was tested in biofluids for both pre‐labeled and label‐free/direct detection of a biologically relevant molecule, demonstrating their versatility. Moreover, the disassembly encoded within the aggregates' design was investigated by utilizing endogenous biomolecules to trigger breakdown. Disassembly in intra‐ and extracellular fluids was further explored to highlight the suitability of AuNP:CB[*n*]:PEG aggregates' use in vivo and eventual clearance from the body through standard physiological routes. The ability to tune disassembly timescales enables these aggregates to be tailored for diverse (bio)applications—from rapid diagnosis to long‐term monitoring—further supporting their potential for future in vivo use.

## 2 Results and Discussion

### 2.1 Self‐Assembly and Kinetic Arrest

**Figure 1:**
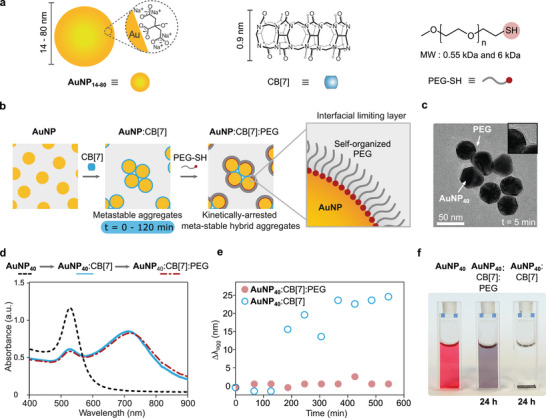
a) Nanoparticle and molecular building blocks used in this study. b) A schematic representation of the kinetic arrest of emerging AuNP:CB[7] aggregates and stabilization of plasmonic hybrids stabilized by interfacial layer of thiolated poly(ethylene glycol) (PEG‐SH) ligands. c) Representative TEM micrograph of AuNP_40_:CB[7]:PEG_6*k*_ aggregates. d) A typical UV–Vis spectra of monodisperse AuNP_40_ (dashed black), AuNP_40_:CB[7] aggregates after 5 minutes of CB[7]‐mediated assembly (solid blue), and the same system immediately after adding PEG_6*k*_ to form AuNP_40_:CB[7]:PEG_6*k*_ hybrid aggregates (dashed red). e) The shift in the plasmon peak of AuNP_40_:CB[7]:PEG_6*k*_ (red circles) assemblies after the addition of PEG_6*k*_ and the AuNP_40_:CB[7] (blue circles) assemblies after 5 minutes of aggregation monitored over 600 minutes. f) A purple‐colored suspension of the AuNP_40_:CB[7]:PEG_6*k*_ aggregates after 24 hours compared to the AuNP_40_:CB[7] aggregates that precipitated and the red‐colored suspension of AuNP_40_.

Kinetic entrapment of plasmonic aggregates implemented both synthesized and commercially available citrate‐stabilized AuNPs (14 nm (prepared in‐house) = AuNP_14_; 20, 40, 60, and 80 nm (BBI) = AuNP_20, 40, 60, 80_) (Figure 1a and Figure S1), which exhibit well‐established optical properties and strong binding affinity to CB[*n*] species.^[^ ^44^, ^45^ ^]^ To kinetically arrest the AuNP:CB[7] aggregates, thiol functionalized poly(ethylene glycol) (PEG‐SH) species (Figure 1a, molecular weight (MW) = 0.55 kDa (PEG_0.55*k*_) and 6 kDa (PEG_6*k*_)) were selected on account of their established affinity for the Au surface.^[^ ^46^ ^]^ Self‐assembly was triggered through the addition of CB[7] to a suspension of AuNPs in water. Kinetic arrest was then achieved through the addition of a PEG‐SH solution (Figure 1b) resulting in the construction of a self‐organized PEG‐SH shell. The formation of colloidally stable AuNP:CB[7]:PEG aggregates was comprehensively studied by UV–Vis spectroscopy (UV–Vis), dynamic light scattering (DLS), electrokinetic ζ potential, and transmission electron microscopy (TEM) experiments. A typical morphology of the resulting AuNP_40_:CB[7]:PEG_6*k*_ hybrids was visualized using TEM (Figure 1c; Figure S2, revealing quasi‐fractal AuNP_40_:CB[7]:PEG aggregates with an average diameter of ca. 330 nm (Figure S3). These resulting quasi‐fractal structures can be attributed to the rapid aggregation process falling within a diffusion‐limited colloidal growth (DLCA) regime, which has previously been reported.^[^ ^19^ ^]^

In Figure 1d, AuNP_40_ was chosen to model PEG‐assisted kinetic arrest on account of their clear distinction between the absorption bands characteristic of the localized plasmon resonance (LSPR) of individual particles at 526 nm and the plasmonically coupled resonance of AuNP_40_:CB[7]:PEG aggregates between 680–900 nm (long chain modes). The evolution of aggregation was reflected in the progressive decrease in the LSPR band intensity and the subsequent appearance and growth of long‐chain modes (Figures 1d and S4). An injection of an aqueous PEG_6*k*_ solution (i.e., 800 PEG‐SH chains per NP; grafting density, ρ = 0.166 PEG nm^−2^, vide infra) into the AuNP_40_:CB[7] suspension during self‐assembly lead to the spontaneous arrest of growth, trapping the AuNP_40_:CB[7] aggregates within a PEG‐SH shell. Building on previous findings that highlighted importance of the time‐controlled aspect of this self‐assembly method, the AuNP_40_:CB[7] aggregates were allowed to form for ca. 5 minutes before PEG‐SH was introduced to ensure the reproducibility across batches.^[^ ^47^ ^]^ The kinetic arrest was evident by the consistent absorbance values at 526 and at 720 nm and no continuous red‐shift in the coupled plasmon band, Figure 1e. An initial red‐shift in the coupled mode resonance was observed immediately after the addition of the PEG‐SH, which is a direct indication of the successful attachment of AuNPs surfaces as a result of a higher‐refractive index (RI) organic PEG‐SH layer (RI_*PEG*_ = 1.4) (Figure S5).^[^ ^48^ ^]^ Further confirmation that PEG‐SH was attached to the surface of the aggregates was observed through the decrease in negative surface charge of the AuNP_40_:CB[7]:PEG_6*k*_ to ca. ζ = −9 mV in comparison to AuNP_40_:CB[7] aggregates (ζ = −24 mV) and monomeric AuNPs_40_ (ζ = −37 mV), Figure S6. Continuous growth of non‐arrested AuNP_40_:CB[7] aggregates was noted via the evolution (i.e., red‐shift) of the absorption band at 720 nm to longer wavelengths. The AuNP_40_:CB[7] assemblies showed eventual precipitation with no reversible character (Figure 1f). We note that some precipitation of the larger AuNP:CB[n]:PEG aggregates could be observed, but the particles were resuspended by gentle shaking – a similar behavior which has been reported by Weinstock and coworkers with AuNP supraspheres.^[^ ^49^ ^]^ The PEG‐assisted arrest was shown to be applicable to all sizes of AuNPs 14 to 80 nm (Figure S7 and Table S2) as well as differing PEG‐SH MWs (0.55 and 6 kDa; 2 kDa data in Supporting Information) showcasing the broad adaptability and tunability of this process.

### 2.2 Biofluids and SERS

In water, long‐term monitoring of the AuNP:CB[7] aggregates kinetically arrested with 0.166 PEG nm^−2^ (ρ_*opt*_) showed improved stability on the order of days to weeks compared to tens of minutes for AuNP:CB[7] aggregates alone (Figure S8). More complex biological solutions; however, can affect the stability of the aggregates. High salinity is known to irreversibly aggregate metallic AuNPs, leading to coalescence. Proteins passivate the surface, reducing accessibility. For these reasons, the stability of AuNP_40_:CB[7]:PEG aggregates in classical buffers (1X phosphate‐buffered saline), simulated urine (SU), simulated cerebrospinal fluid (SCSF), and a protein‐rich media (i.e., fetal bovine serum (FBS)) was evaluated to assess the suitability for biomedical applications. In the presence of all biofluids, the optical properties of AuNP_40_:CB[7]:PEG_0.55*k*_ were significantly affected (Figure S9), indicated by the decrease in absorbance of the total spectra. Some spectra shape was partially maintained in 1X PBS after 5 hours, but after 24 hours all spectra shape was lost. Even in the presence of FBS the spectra of the AuNP_40_:CB[7]:PEG_0.55*k*_ eventually flattened suggesting either additional aggregation or adsorption of the particles to the container surface.^[^ ^50^ ^]^

In contrast, in Figure S10, AuNP_40_:CB[7]:PEG_6*k*_ aggregates show a much less severe decrease in absorption or a slight blue‐shift of the LSPR when exposed to the biofluids, which may also arise from the rearrangement of the aggregates. These observations suggest that longer PEG‐SH chains are more suitable for better stability in a wider range of biofluids. Likely a change in the surface coverage of the longer chain PEG‐SH would improve the stability of the aggregates in the higher salinity solutions, but to allow for maximum surface availability for the purpose of SERS sensing the moderate coverage was maintained.

**Figure 2:**
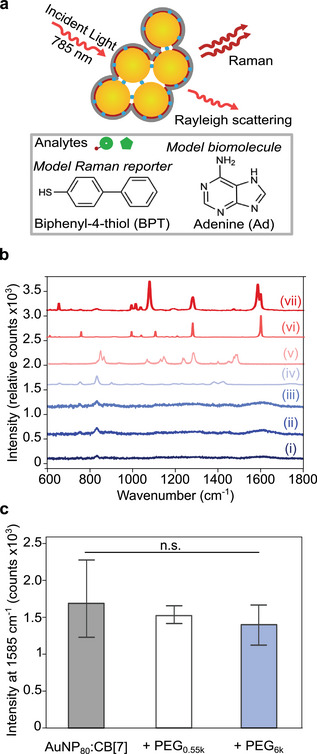
a) Illustration of surface‐enhanced Raman spectroscopy (SERS) with a common Raman reporter, biphenyl‐4‐thiol (BPT), and a bioanalyte, adenine (Ad). b) SERS spectral evolution of (i) AuNP_80_, (ii) AuNP_80_:CB[7], (iii) AuNP_80_:CB[7]:PEG_6*k*_, and (vii) AuNP_80_:CB[7]:PEG_6*k*_ + 1 μM BPT with reference Raman spectra of (iv) CB[7], (v) PEG‐SH, and (vi) BPT. c) Comparison of SERS intensities of 1 μM BPT with AuNP_80_:CB[7], AuNP_80_:CB[7]:PEG_0.55*k*_, AuNP_80_:CB[7]:PEG_6*k*_. Significance was determined using a one‐way ANOVA between arrested aggregates and the AuNP:CB[7] aggregates *n* = 3.

Facile formation of aggregates arrested with 6 kDa PEG‐SH that demonstrate sufficient stability in complex media is important for SERS applications, as the presence of other components in the sensing environment often hinders reliable SERS detection of target biomarkers.^[^ ^24^ ^]^ For SERS application, although a range of AuNP sizes were able to detect a common Raman reporter, biphenyl‐4‐thiol (BPT), AuNP_80_:CB[7]:PEG_6*k*_ aggregates were highlighted as they showed the best sensitivity (Figures S11 and S12), which is supported by previous literature.^[^ ^39^, ^51^ ^]^ The appearance of the SERS signal from BPT at 1585 cm^−1^ peak (characteristic peak of the ring breathing mode for BPT^[^ ^52^ ^]^) as well as CB[7] at 755 cm^−1^ and PEG‐SH at 1479 cm^−1^ (Figure 2b) confirms the presence of these components. No BPT signal detected using only AuNP_80_ confirms that SERS enhancement is arising from the aggregates (Figure S13). In Figure 2c, after the addition of BPT to AuNP_80_:CB[7] aggregates kinetically arrested with PEG_0.55*k*_ and PEG_6*k*_ (and 2 kDa in Figure S14) no significant difference in SERS intensity was observed compared to aggregates with no kinetic arrest in water. This suggests that chain length of PEG‐SH does not alter the permeability of the system. Label‐free direct detection of adenine (Ad), a main building block of DNA and previously identified biomolecule,^[^ ^53^ ^]^ was also investigated through the addition of Ad to AuNP_80_:CB[7]:PEG_*y*_ (*y *= 0.55k and 6k) aggregates and further indicates that the aggregates remain permeable to a biomarker with a lower Au affinity (Figure S15).

**Figure 3:**
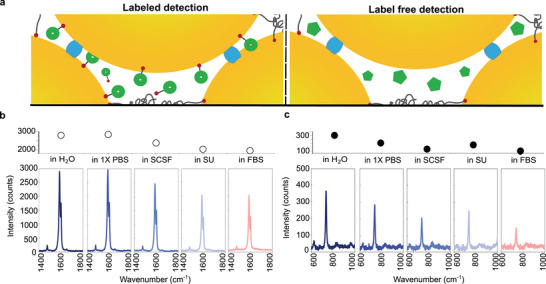
SERS spectra and peak intensities depicting a) Illustration of labeled detection of biphenyl‐4‐thiol (BPT) (left) and label free detection of adenine (Ad) (right) within the interstitial spaces of the AuNP:CB[7]:PEG aggregates. b) labeled detection using 1 μM of BPT at 1585 cm^−1^ and c) label‐free detection of 2 mM of Ad at 734 cm^−1^ in water, 1X phosphate‐buffered saline (1X PBS), simulated cerebrospinal fluid (SCSF), simulated urine (SU), and fetal bovine serum (FBS).

For biosensing, two different sensing approaches are often employed; label‐based (indirect) and label‐free‐based (direct). By pre‐labeling the aggregates with BPT, information about how the environment affects the SERS of the arrested aggregates can be extrapolated versus the influence on the permeability of the system through direct detection of free Ad. The characteristic BPT signal (1585 cm^−1^) remains observable (Figure 3a and Figure S16 for full spectra) at a concentration of 1 µΜ under all conditions and there is no significant decrease in the BPT signal when mixed with 1X PBS compared to water. This suggests that other components in SCSF, SU, or FBS, especially the larger proteins, have a greater impact on the SERS signal than simple ions and that these can affect both the AuNP surface and the reporter, even with the PEG‐SH attached. Interestingly, when the same AuNP_80_:CB[7]:PEG_6k_ aggregates are introduced to the biofluids with free Ad (2 mM) for direct detection the effects are different (Figure 3b and Figure S17 for full spectra). Unsurprisingly, the presence of the FBS reduced the signal of Ad likely due to the formation of the soft protein corona, whereas for the BPT signal this decrease could be due to an increase in light scattering caused by the protein corona. Interestingly, the SERS intensity of the Ad signal decreased more in the SCSF, whereas BPT peaks were reduced in the SU solution, where urea concentrations are immensely overwhelming.

To improve the signal enhancement, higher grafting densities and higher concentrations of aggregates could be employed as well as longer acquisition times. Despite some biofluid effects on the SERS enhancement of AuNP:CB[7]:PEG system, the analytes remain detectable, demonstrating the system's potential for rapid detection in complex environments The facile design of these aggregates further circumvents the need for time‐consuming and costly additional sample processing steps such as filtration and/or dilution and allows for future modifications for long term detection and monitoring.

### 2.3 Disassembly

**Figure 4:**
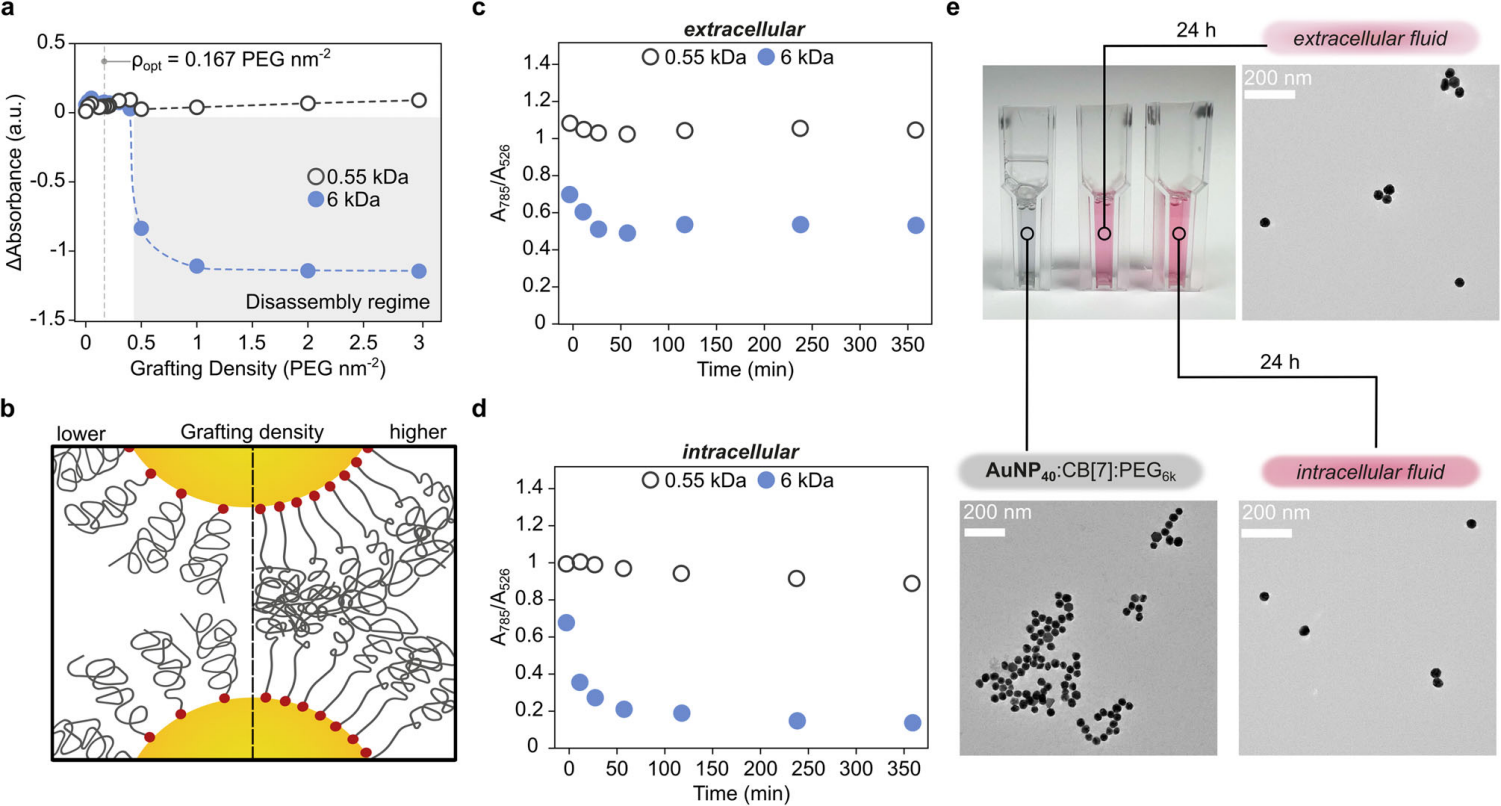
a) Disassembly regime of the AuNP_40_:CB[7]:PEG_*x*_ aggregates, where x = 0.55k and 6k, determined by the MW of the PEG‐SH and grafting density. ΔAbsorbance (a.u.) = [t_59_(A_785*nm*_/A_526*nm*_) ‐ t_0_(A_785*nm*_/A_526*nm*_)] x dilution factor. b) Change in PEG‐SH conformation on a curved surface with an increase in grafting density concentrated polymer brush (CPB) conformation and a semi‐dilute polymer brush (SDPB) conformation. The ratio of A_785_/A_526_ of the AuNP_40_:CB[7]:PEG_6*k*_ when exposed to c) extracellular and d) intracellular media over time. e) Representative TEM images with photo of cuvettes depicting the aggregates 24 hours before and after exposure to intracellular and extracellular media.

For the use of aggregated nanoparticles in vivo, a crucial parameter that must be considered is whether the aggregates can disassemble for eventual clearance. It is especially important that aggregates comprised of large NPs encompass the ability to disassemble to increase the probability of clearance from the body. In the design of the AuNP_40_:CB[7]:PEG aggregates, disassembly of the resulting aggregates (Figure 4a) can be triggered by excess PEG‐SH. Stability was monitored over time with different grafting densities and PEG‐SH MWs to assess whether an increase of the LSPR peak and decrease of the 785 nm peak (Figure S18) occurs simultaneously, which would be indicative of the disassembly of the AuNP_40_:CB[7]:PEG_*x*_ (where x = 0.55k, 2k, and 6k; all 2k data in the supplemental information) aggregates. Disassembly was found to be both dependent on grafting density and MW/chain length of the PEG‐SH ligands, where aggregates arrested with 0.55 kDa PEG‐SH ligands did not disassemble even at high PEG‐SH grafting densities evaluated (3 PEG nm^−2^ = 1.669 mM), but disassembly of AuNP_40_:CB[7]:PEG_6*k*_ aggregates occurred at 0.5 PEG nm^−2^ = 0.278 mM. These results suggest the “optimal grafting density” for the AuNP:CB[7]:PEG system to be ρ_*opt*_ = 0.166 PEG nm^−2^ and was defined as the median number of PEG‐SH chains per nm^2^ of AuNP surfaces required to afford stabilization of AuNP:CB[7] aggregates for at least 60 minutes for all PEG‐SH MWs.

Previous reports utilizing a physiological thiol to trigger disintegration of Au clusters mainly attributed the disassembly to the change in pH as they observed no disaggregation at high thiol concentrations without a pH adjustment.^[^ ^42^ ^]^ In contrast, disassembly of the AuNP_40_:CB[7]:PEG_6*k*_ aggregates was observed without any pH adjustments, which suggests an alternative disaggregation mechanism. Although displacement of CB[7] by excess PEG‐SH ligands could contribute, if the displacement of CB[7] was the sole cause of disassembly, then the kinetics in Figure 4a and Figure S19 would overlap and the minimum grafting density threshold would be consistent regardless of the PEG‐SH MW, which is not observed. As a result, another potential mechanism can be considered based on the changes in the free energy at the surface of the AuNPs and the repulsive interactions between the grafted polymers due to a change in PEG‐SH chain conformations.

It is well reported that PEG‐SH chains organized on flat metallic interfaces can adopt either a collapsed “mushroom” or an extended “brush‐like” conformation.^[^ ^54^, ^55^ ^]^ In the brush‐like conformation PEG‐SH chains are extended outward at a higher packing density, while a mushroom conformation results from loose packing wherein the PEG‐SH chains are partially collapsed on the surface.^[^ ^26^, ^56^ ^]^ Therefore, when two flat surfaces modified with extended PEG‐SH chains come within a certain distance from one another, there can be repulsion due to the tangling of the PEG‐SH chains.^[^ ^57^, ^58^ ^]^ Shorter chains require a much closer distance to experience intertwining of the chains.^[^ ^59^, ^60^, ^61^ ^]^

Polymers grafted to spherical NPs experience a similar change in polymer conformation depending on PEG‐SH chain length and grafting density. Similar to a flat surface, increasing the grafting density of PEG‐SH chains forces an extension of the polymer chains from a concentrated brush polymer conformation (CPB) to a semi‐dilute polymer brush conformation (SDPB).^[^ ^62^ ^]^ In contrast to a flat surface, the curvature of the NP surface can also determine the point (i.e., the critical radius) at which the conformations of the PEG‐SH chains transition from a CPB to SDPB conformation.^[^ ^63^ ^]^ Specifically, a higher degree of curvature (smaller NP size) forces the polymer chains to extend further from the NP surface on account of an increase in radial space or free volume further from the surface, which results in an extended conical shape and more of the polymer chain overlap increasing steric repulsion, Figure 4b.^[^ ^64^ ^]^ This behavior is supported in Figure S20 by observing a larger degree of disassembly for the smaller AuNP_20_:CB[7]:PEG_6*k*_ aggregates at comparable grafting densities than that of AuNP_40_:CB[7]:PEG_6*k*_ and AuNP_80_:CB[7]:PEG_6*k*_ aggregates. Therefore, the tendency for AuNP:CB[7]:PEG aggregates to disassemble with PEG_6*k*_, can be explained by either (i) displacement of CB[7] by excess PEG‐SH ligands and/or (ii) a higher propensity of longer PEG‐SH chains to transition from SDPB to CPB, which leads to a significantly higher free energy of steric repulsion between localized PEG‐SH layers between neighboring particles.^[^ ^57^, ^65^, ^66^ ^]^ A deeper mechanistic investigation could provide valuable insights to expand the use of these aggregates.

Notably, in addition to PEG‐SH triggering disassembly, thiolated biological molecules such as cysteine (Cys) and glutathione (GSH) were also found to cause disassembly of the aggregates. At the biological concentration of 5 µM of Cys (3.58 × 10^5^ Cys molecules per nm^2^), the aggregates did not undergo immediate disassembly (Figure S21). Whereas, at a 200 µM concentration of Cys (1.43 × 10^7^ Cys molecules per nm^2^) the aggregates underwent immediate disaggregation. Similar trends were seen with GSH in Figures S22–S24. In contrast, ethanol, a non‐thiolated molecule, did not induce disassembly (Figure S25), suggesting that functionality may contribute to the disassembly mechanism. Since the addition of small thiolated molecules will not pack in the same manner as excess PEG‐SH to change the conformation, shifts in the ligand density causing a local conformational change may also trigger disassembly.^[^ ^67^ ^]^ These findings suggests that thiol‐containing molecules with appropriate structural features and interactions with PEG‐SH ligands can destabilize the AuNP_40_:CB[7]:PEG_6*k*_ aggregates, and extent of disassembly likely depends on both thiol concentration and molecular characteristics.

Stabilization of the aggregates was observed when AuNP_40_:CB[7]:PEG_6*k*_ aggregates were exposed to other biological components such as bovine serum albumin (BSA) or cystine (Cys–Cys) (Figures S26 and S27) prompting additional evaluation in intracellular and extracellular media. In extracellular media, AuNP_40_:CB[7]:PEG aggregates for all PEG‐SH MWs did not fully disassemble (Figure 4c), as indicated by the plateau reached at 60 minutes. In contrast, complete disassembly of the AuNP_40_:CB[7]:PEG_6*k*_ aggregates was reached in intracellular media after 90–100 minutes, defined by the absorbance ratio falling near or below 0.1 in Figure 4d. AuNP_40_:CB[7]:PEG_0.55*k*_ aggregates exhibited the lowest degree of disassembly in both media, showing incomplete disassembly even after 6 hours, demonstrating the tunability of these aggregates through facile changes of the PEG‐SH MW (Figures S28 and S29c,d for 2 kDa data). However, the AuNP_40_:CB[7]:PEG_6*k*_ aggregates in intracellular media underwent a similar disassembly as the AuNP_40_:CB[7]:PEG_0.55*k*_ aggregates. Noting that the concentration of thiolated molecules, i.e., Cys and GSH, are one and two orders of magnitude higher in intracellular media than extracellular media (Table S3), respectively,^[^ ^68^, ^69^ ^]^ a higher degree of disassembly in intracellular media is not unexpected and suggests that the presence of the BSA and Cys–Cys do not completely inhibit disaggregation.

## 3 Conclusion

Herein, we report a rapid and facile method to prepare kinetically arrested metastable hybrid SERS‐active nanoaggregates comprised of metastable AuNP:CB[7] aggregates kinetically arrested with a self‐assembled thiolated PEG‐SH shell. The resulting assemblies are tunable through modulating AuNP size (14–80 nm) and PEG‐SH MW (0.55 and 6 kDa) providing access to customized SERS substrates pertinent to a wide range of applications. Stability of the the hybrid aggregates can be tuned and extended through ratiometric adjustment of the PEG‐SH to AuNP surface area and PEG‐SH chain length. Detection of a model Raman reporter BPT within AuNP:CB[7]:PEG aggregates at concentrations as low as one micromolar was possible by the rigid sub‐nanometer gaps within the aggregates on account of the CB[7] molecular spacers. Additionally, both BPT and Ad were also readily detectable in a range of biofluids, showcasing the ability of AuNP:CB[7]:PEG nanoaggregates to overcome the challenges of high salinity and non‐specific protein binding in biosensing. Moreover, BPT and Ad were found to readily permeate through the PEG‐SH shell on the aggregates, highlighting the balance achieved in the PEGylated aggregates between enhanced stability and SERS sensitivity. Moreover, incorporation of an encoded disassembly mechanism within the AuNP:CB[7]:PEG aggregate design supports potential in vivo application, where stability is required for delivery to the targeted location and subsequent triggered disassembly is essential for clearance following diagnosis. The tunable nature of the AuNP:CB[7]:PEG aggregate and stability in a wide range of biofluids and permeability to (bio)analytes at low concentrations ensures their suitability for a wide range of potential uses encompassing both rapid biosensing and long‐term monitoring for future in vivo applications.

## 4 Experimental

All experimental details are outlined within the Supporting Information.

## Conflict of Interest

The authors declare no conflict of interest.

## Supporting information

Supporting Information

## Data Availability

The data that support the findings of this study are available from the corresponding author upon reasonable request.
