# Supplementary material for: Kinetically Arrested SERS‐Active Aggregates for Biosensing
Source: Chemistry. 2025 Jun 17;31(38):e202500915. doi: 10.1002/chem.202500915 (PMC12238919; doi:10.1002/chem.202500915)
Supplement: Supplementary file 1 — Supporting Information [file CHEM-31-e202500915-s001.pdf]

Kinetically Arrested SERS-Active Aggregates for Biosensing

Natalie S. Potter[1], Kamil Sokołowski[1], Renata Lang Sala[1], Jade A. McCune[1], and Oren A. Scherman*[1]

[1] *Melville Laboratory for Polymer Synthesis, Yusuf Hamied Department of Chemistry, University of Cambridge, Cambridge, CB2 1EW, United Kingdom*
[*] *e-mail: oas23@cam.ac.uk*

S.1 Materials

All materials as follows were purchased from commercial suppliers and used without further purification unless otherwise stated: Gold (III) chloride trihydrate ($HAuCl_4$•$3H_2O$, 99.9%; Sigma-Aldrich), trisodium citrate dihydrate (>99.5%), and poly(ethylene glycol) methyl ether thiol (PEG-SH) (6 kDa and 2 kDa, Sigma-Aldrich and 0.55 kDa, BOC Sciences:PEG Solutions). Citrate coated gold nanoparticles (AuNPs) with a reported hydrodynamic radius (D_h) of 20, 40, 60 and 80 nm were purchased from BBI Solutions and used as received. HPLC grade water was used in all aqueous solutions, all glassware was cleaned with *aqua regia* before experiments.

Cucurbit[7]uril (CB[7]) was prepared and purified using previously reported methods.[1,2] Simulated cerebrospinal fluid (SCSF) and simulated urine (SU) were prepared using previously published protocols.[3]

S.2 Methods

S.2.1 Synthesis of $AuNP_{14}$

$AuNP_{14}$ were synthesised using a modified Turkevich method.[4,5] In brief, a solution of $HAuCl_4$•$3H_2O$ (50 mg/mL in HPLC grade water; 0.127 mol) in 99 mL of Millipore water was heated to reflux for at least 30 min. Under vigorous stirring a solution of trisodium citrate dihydrate (224 mg/mL in HPLC grade water; 0.762 mol) was added quickly at 100 °C and the reaction system was cooled down to 80 °C and held at this temperature for the next 2 h. Then the solution was cooled in an ice bath for 30 min. A clear dark-red solution was obtained and stored at 4 °C for further use. Nanoparticle size was estimated to be 16 ± 0.1 nm (DLS) and 12 ± 1.4 nm (TEM, n = 152).

S.2.2 Formation of AuNP:CB[7]:PEG aggregates

For the formation of AuNP:CB[7] aggregates, one volume of CB[7] (0.5 mM) was deposited to the bottom of a cuvette. For 14, 20, 40, 60, and 80 nm AuNPs, CB[7] volumes of 7, 8, 10, 20, and 40 μL were used, respectively. 2.5 mL of AuNP stock solution was added to the cuvette and allowed to assemble for 5 minutes or until a colour change occurred. The degree of assembly was monitored *via* UV-Vis spectroscopy. After ca. 5 min, a volume of poly(ethylene glycol) thiol PEG-SH (0.05 mM - 0.55 kDa, 2 kDa or 6 kDa) was added and the cuvette was gently shaken to thoroughly disperse the PEG-SH throughout the CB[7]-AuNP sample. The volume of PEG-SH added depended on the desired grafting density. In general, amounts equaling 0.083, 0.166, 0.333, 1, and 2 PEG nm^{-2} were most often used to kinetically arrest the aggregates. The grafting density was calculated based on the total surface area of individual particles in the solution. For all SERS experiments and TEM images, 0.166 PEG nm^{-2} was used to kinetically arrest AuNPs.

Table S1: Example volumes used to form the $AuNP_{40}$:CB[7]:PEG aggregates for 1 mL of monomeric nanoparticles.

Desired grafting density (PEG nm^{-2})	Volume of CB[7] (0.5 mM μL)	Volume of PEG-SH (0.05 mM μL)
0.166	10	1.85
1	10	11.13

S.2.3 Ultraviolet-Visible spectroscopy (UV-Vis)

UV-Vis spectra were obtained using a Varian Cary 50 spectrometer at room temperature unless otherwise noted. For AuNP:CB[7] assembly monitoring and kinetic arrest, samples were prepared in either quartz cuvettes or disposable cuvettes and were baselined using the appropriate container. CB[7]-AuNP assembly and kinetic arrest protocol are outlined in the synthesis section above. For the short term stability experiments, the kinetically arrested CB[7]-AuNPs were measured first before aggregation, during the 5 min, and then 30 min after kinetic arrest.A 96-well plate was used in a CLARIOstar plate reader (BMG Labtech) was used with the CLARIOstar MARS analysis software for long-term stability experiments and PEG grafting density variation experiments.

S.2.4 Dynamic Light Scattering (DLS) and Zeta Potential

Dynamic light scattering measurements were achieved using a Malvern Zetasizer Nano ZS90 fitting with a He-Ne laser (λ = 663 nm) at room temperature. The hydrodynamic diameters (D_h) were obtained based on the Stokes-Einstein equation. Disposable cuvettes were used for DLS measurements and all measurements were taken in water. Zeta potential values were calculated using the Smoluchowski model and folded capillary zeta cells.

S.2.5 Raman Spectroscopy

All SERS measurements were taken using a LabRAM HR Evolution Raman Spectrometer by Horiba Scientific paired with an XTRA II high power single frequency diode laser at 785 nm. SERS measure-

ments were taken from 600 - 1800 cm^{-1} and either a 20 s acquisition time was set with 8 accumulations (for BPT and Ad measurements in water) or a 10 s acquisition time with 5 accumulations (for measurements taken in the biofluids). AuNP:CB[7]:PEG aggregates were prepared fresh the same day before each SERS experiment and measurements were taken immediately after analytes were introduced. A 10x objective was used with a 600 grooves/mm (750 nm) grating and 100% ND filter with a source laser power of ca. 82 mW. SERS spectra were baselined using OriginSoftware before further analysis. For biofluid experiments using Raman reporter 4-biphenyl thiol (BPT), AuNP$_{80}$:CB[7]:PEG$_{6k}$ aggregates were first prepared and labeled with BPT. Subsequently the biofluids (1X phosphate-buffered saline (PBS), simulated cerebrospinal fluid (SCSF), simulated urine (SU), or fetal bovine serum (FBS)) was mixed together in a 50:50 ratio (final concentration of BPT was 1 μM). For the detection of free Adenine (Ad), AuNP$_{80}$:CB[7]:PEG$_{6k}$ aggregates were mixed first with the biofluid of choice (50:50 by volume) and then the analyte of interest was added to meet the final concentration (2 mM Ad) .

S.2.6 Transmission Electron Microscopy

Transmission electron microscopy was carried out using a Talos F200X G2 Scanning Transmission Electron Microscope (S/TEM). For the CB[7]-AuNP samples, both kinetically arrested and not, samples were made the same day of the imaging and were diluted 1:10 before being drop-cast onto a continuous carbon film 300 mesh copper grid.

S.2.7 Extended Discussion: Disassembly Triggered by PEG Repulsion (Planar surfaces)

As observed by Kenworthy *et al.* the repulsion mechanism for higher molecular weight PEG primarily stems from steric pressures whereas for significantly shorter polymer chains (300 – 700 Da) the total repulsion is comparable to that of the electrostatic pressures.[6,7] This was further investigated that the mushroom conformation can bypasses head-on interactions between adjacent chains at lower grafting densities whereas the brush conformation introduces a steric repulsion as a result of interchain interactions. At lower grafting densities of longer chain polymers some interdigitation may be allowed but through an introduction of excess PEG paired with the onset of steric pressure due to the confinement between two or more AuNPs can result in the aggregates being forced apart. This behavior can additionally be described by the free energy of the polymer chains confined between AuNPs. Similarly described by Dolan and Edwards and others, for polymers between two planar surfaces in the mushroom regime at lower grafting density the free energy is low due to the polymers having no lateral interactions with each other as well as little head-on interactions.[8–10] This free energy can be increased either by decreasing the distance between the Au surfaces or by increasing the number of PEG's within a confined space thus increase lateral interactions and as a result the head-on interactions due to extending the polymer chains. For our system, the latter is the most likely due to the rigid CB[7] which allows us to assume no significant change to the distance between the AuNPs within one aggregate is occurring. The curvature AuNP:CB[7]:PEG aggregates could influence both the total steric repulsion between each AuNP within the aggregates and the potential lateral interactions between the PEGs. However, the behavior of the

PEGs solely between the AuNP interfaces should follow a similar free energy model and would suggest that the PEGs are mainly responsible for overcoming the energetic affinity holding the AuNPs together.

S.3 Supplementary Data

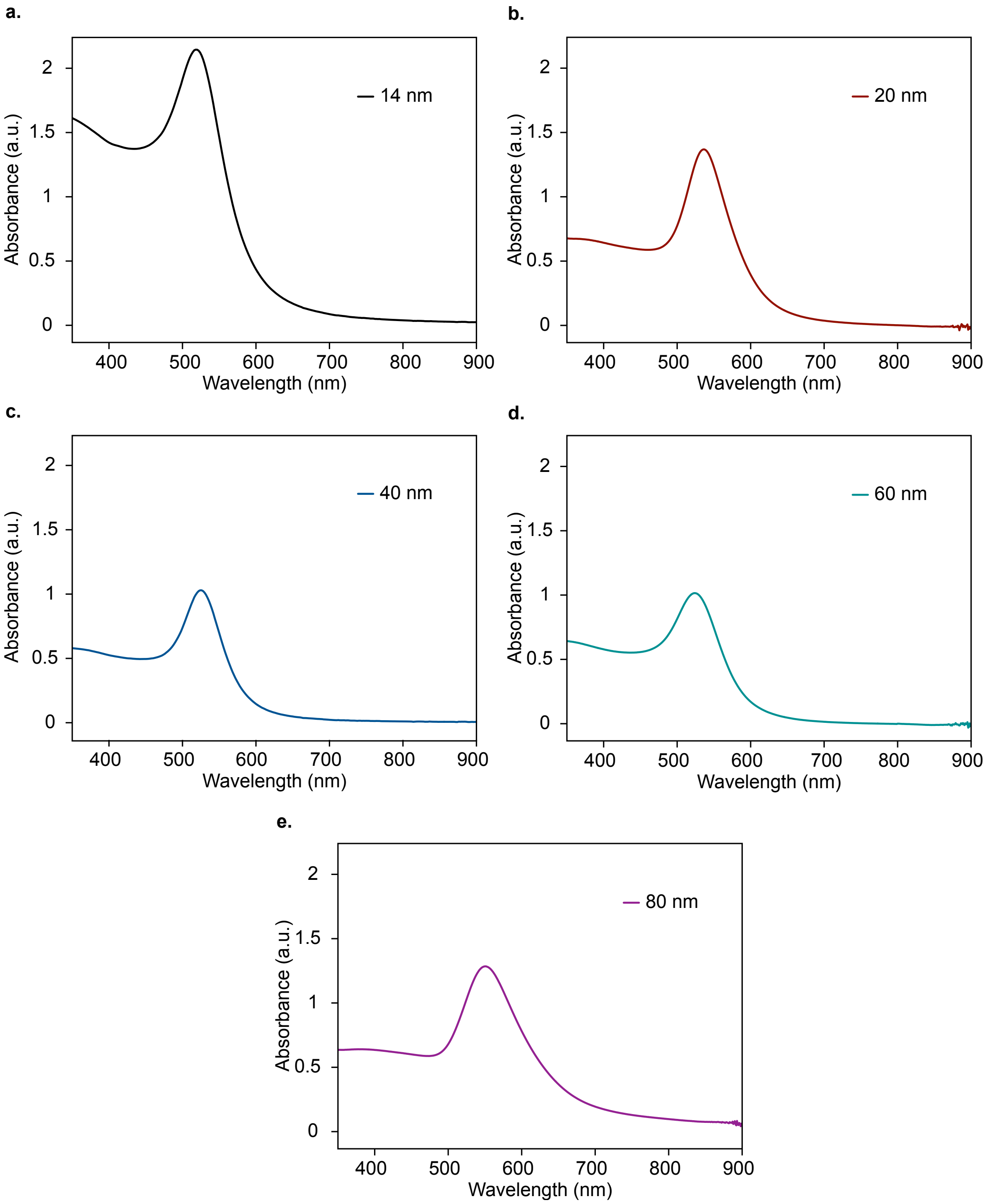

Figure S1: UV-Vis of different sizes of AuNP monomers used throughout **a** 14 nm (in-house synthesis), **b** 20 nm (BBI), **c** 40 nm (BBI), **d** 60 nm (BBI), **e** 80 nm (BBI).

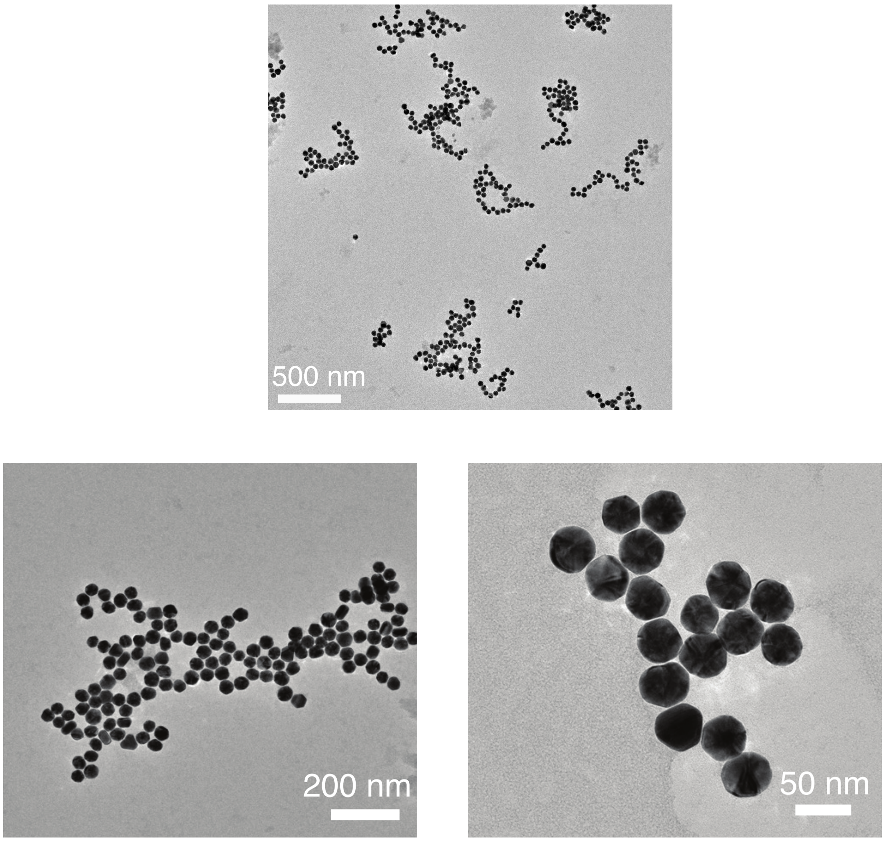

Figure S2: Representative TEM images depicting quasi-fractal $AuNP_{40}$:CB[7]:PEG_{6k} aggregates.

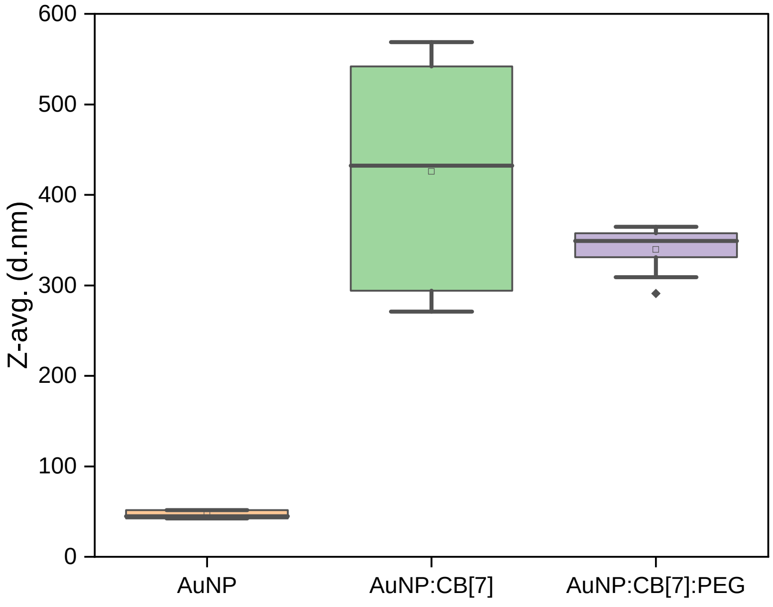

Figure S3: Hydrodynamic diameters measurements of $AuNP_{40}$, $AuNP_{40}$:CB[7] and $AuNP_{40}$:CB[7]:PEG_{6k}. n = 3 for assemblies.

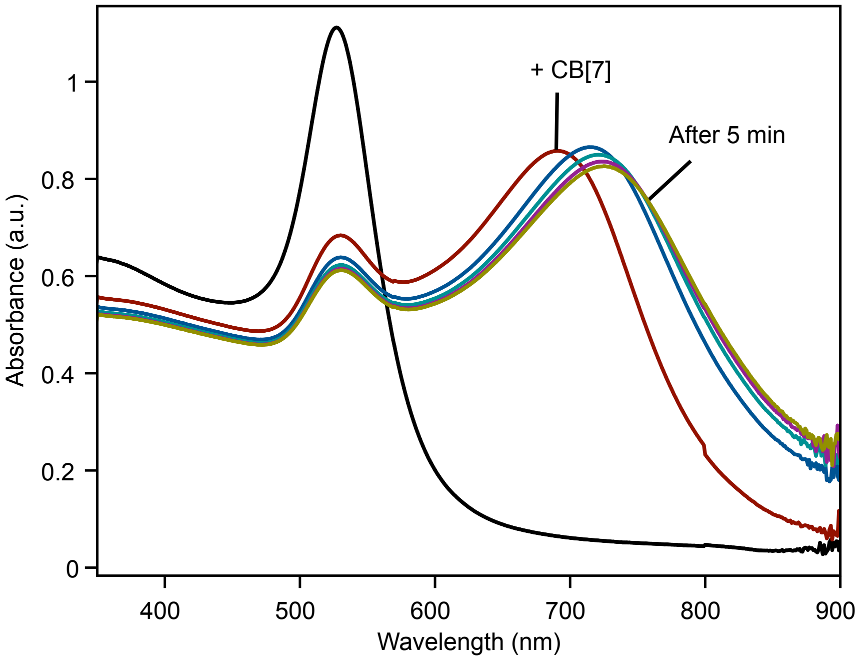

Figure S4: UV-Vis spectra of $AuNP_{40}$:CB[7] aggregation progress. AuNPs were aggregated for 5 min by the addition of CB[7] indicated by growth and red-shift of the secondary absorption peak.

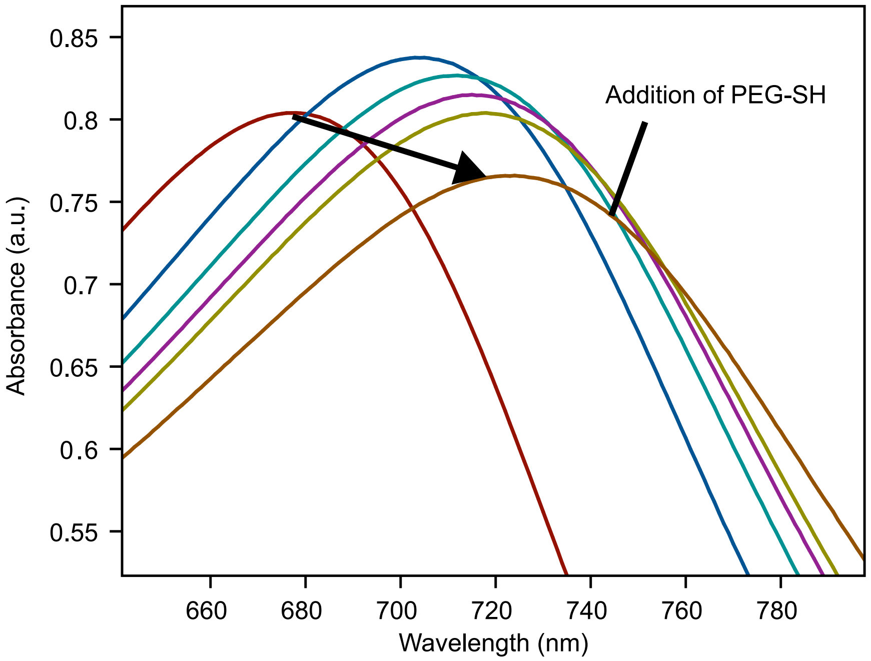

Figure S5: UV-Vis spectra of $AuNP_{40}$:CB[7] aggregates kinetically arrested with PEG-SH (6 kDa) after *ca.* 5 min. Confirmation of the PEG-SH attaching to the Au surface is indicated by an additional red-shift in the absorption band due to the change in refractive index (orange line).

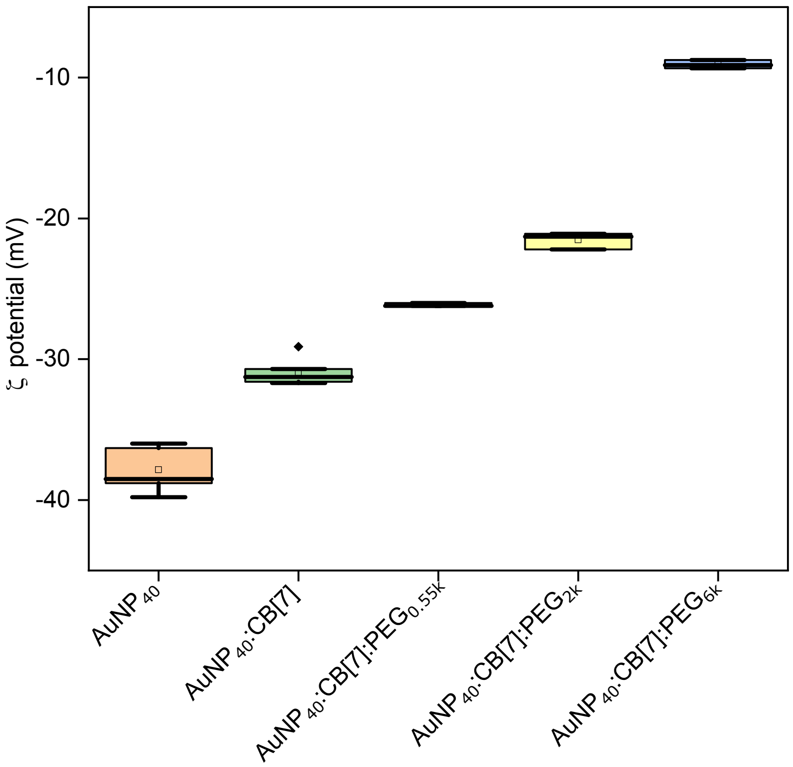

Figure S6: Zeta potential measurements of $AuNP_{40}$, $AuNP_{40}$:CB[7], $AuNP_{40}$:CB[7]:$PEG_{0.55}$, $AuNP_{40}$:CB[7]:PEG_{2k}, and $AuNP_{40}$:CB[7]:PEG_{6k}.

Table S2: Overview of localized surface plasmon resonance (LSPR) band before and after the addition of CB[7] and their resulting aggregate size for monomeric AuNPs of various sizes.

AuNP Size (nm)	LSPR (nm)	LSPR peak after CB[7] addition (nm)	Aggregate size (d.nm)
14	518	634	493 ± 155
20	524	648	631 ± 64
40	526	720	329 ± 25
60	537	813	163 ± 12
80	548	854	242 ± 46

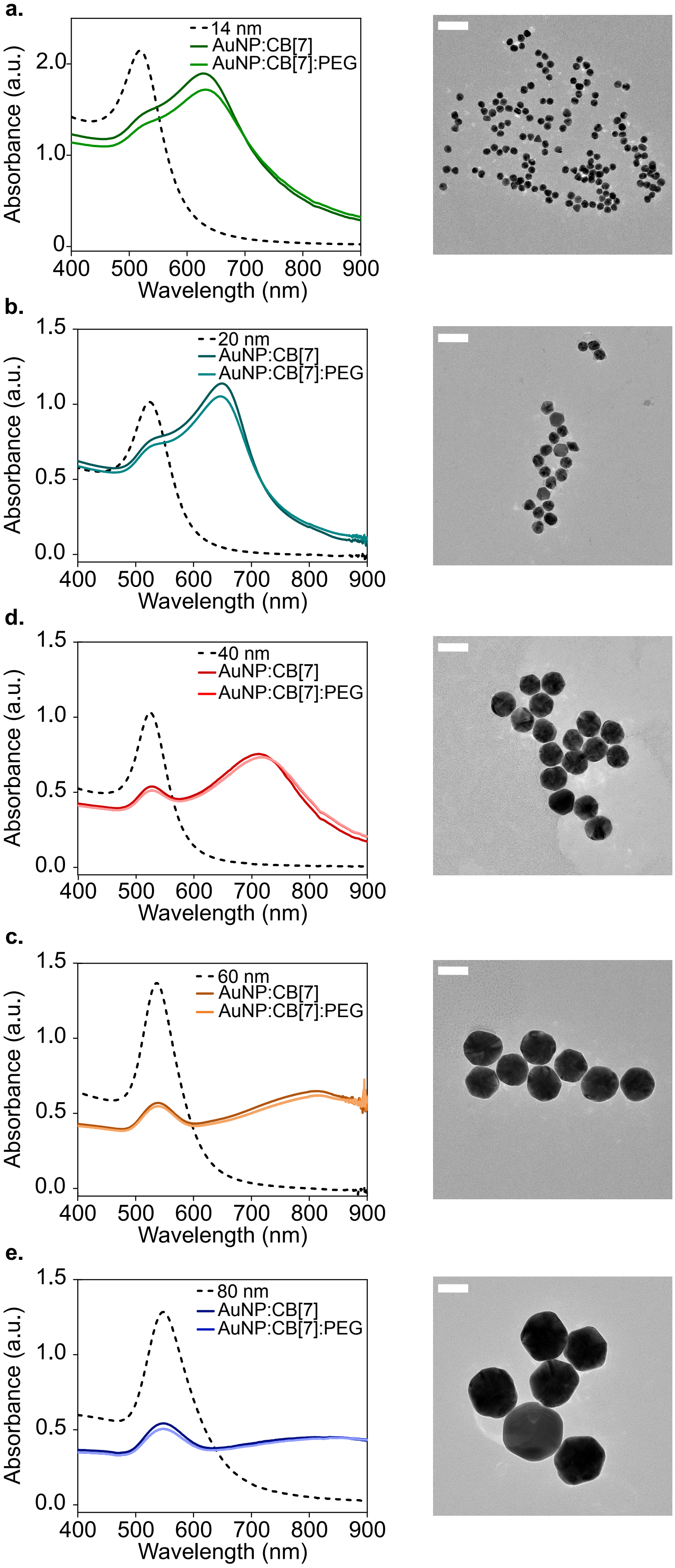

Figure S7: Aggregation of **a.** 14 nm, **b.** 20 nm, **c.** 40 nm, **d.** 60 nm, and **e.** 80 nm AuNPs with CB[7] and kinetically arrested with PEG_{6k} monitored via UV-Vis and representative TEM micrographs of the resulting aggregates. Scale bar = 50 nm.

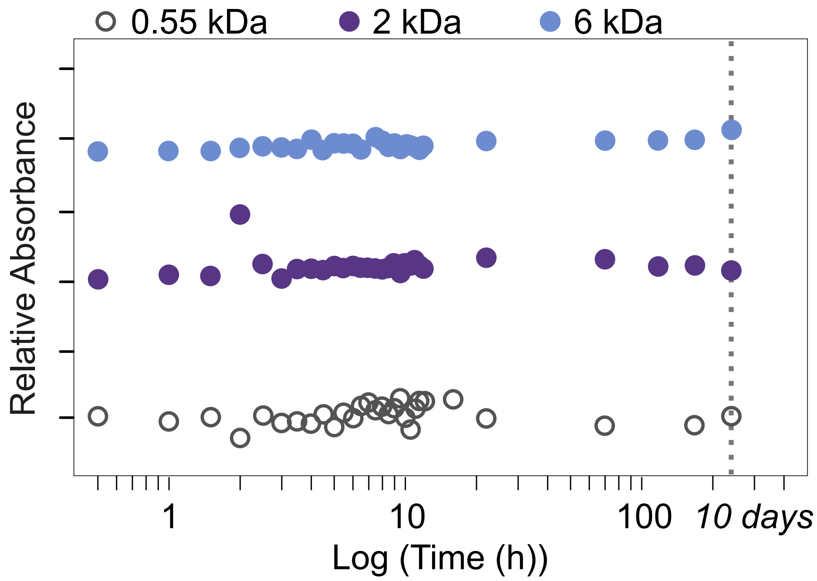

Figure S8: Long term stability of $AuNP_{40}$:CB[7]:PEG_x aggregates in water, where x = 0.55k, 2k, and 6k

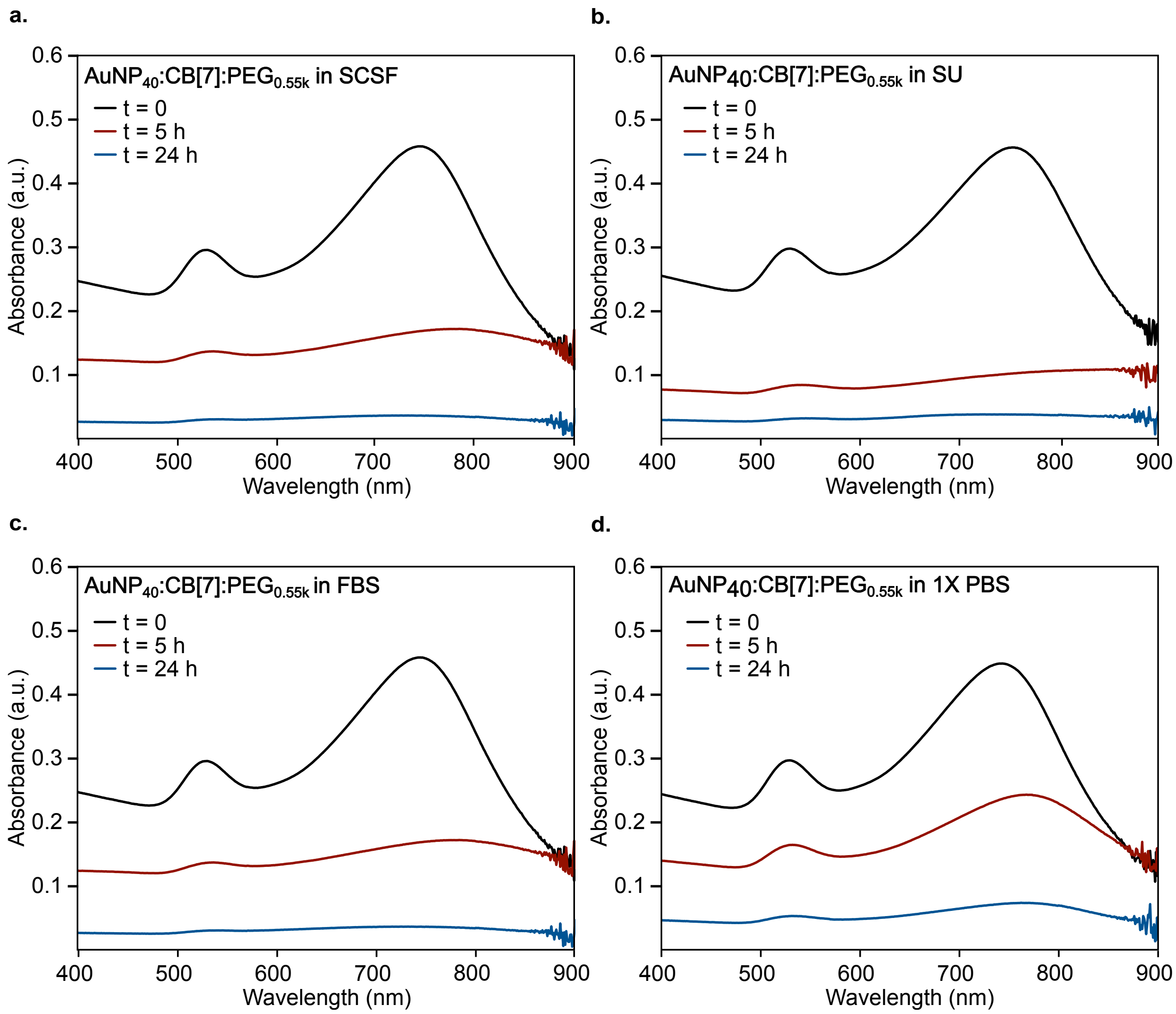

Figure S9: UV-Vis of AuNP$_{40}$:CB[7]:PEG$_{0.55k}$ in **a.** simulated cerebrospinal fluid (SCSF), **b.** simulated urine (SU), **c.** fetal bovine serum (FBS), and **d.** 1X phosphate-buffered saline (PBS) for 0 h, 5 h, and 24 h

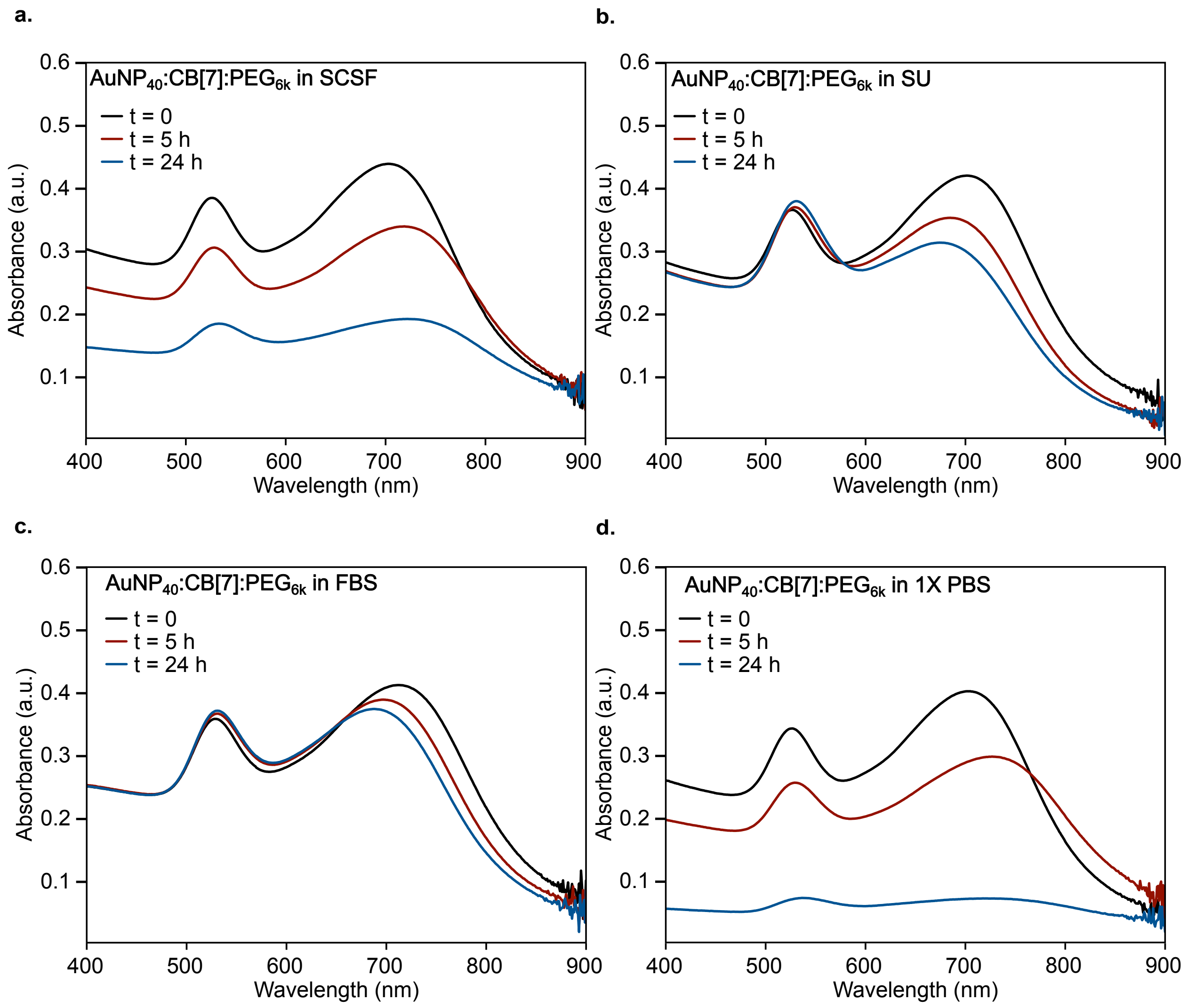

Figure S10: UV-Vis of $AuNP_{40}$:CB[7]:PEG_{6k} in **a.** simulated cerebrospinal fluid (SCSF), **b.** simulated urine (SU), **c.** fetal bovine serum (FBS), and **d.** 1X phosphate-buffered saline (PBS) for 0 h, 5 h, and 24 h.

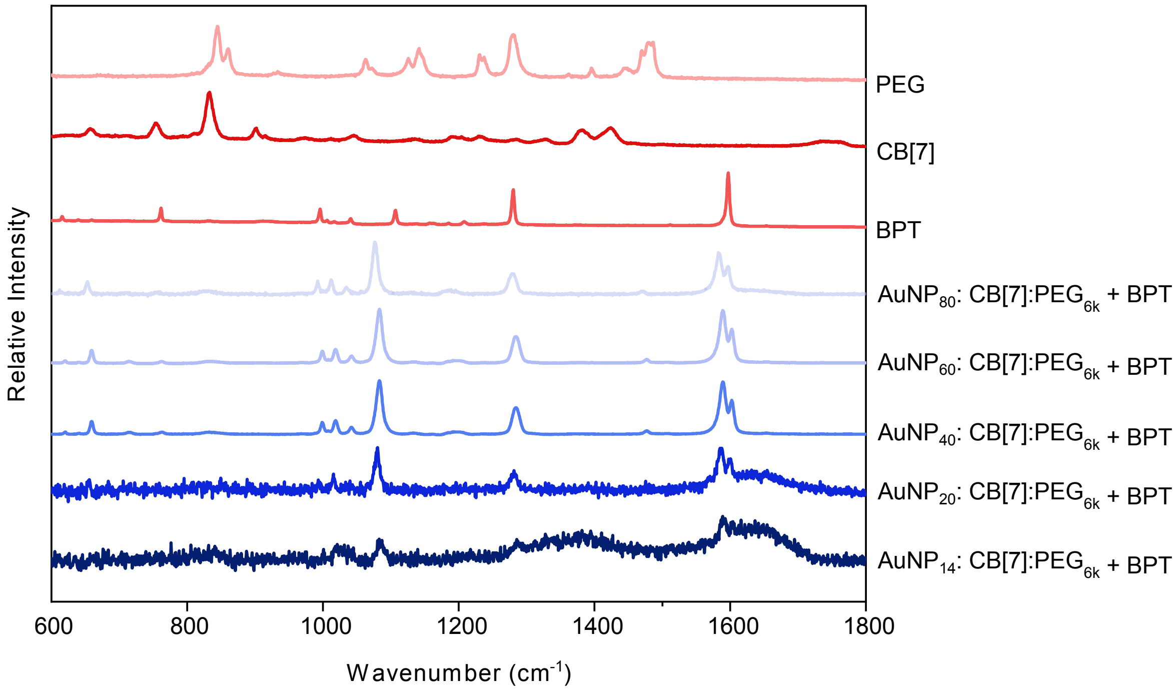

Figure S11: Full SERS spectrum of $AuNP_x$:CB[7]:PEG_{6k} (where x = 14, 20, 40, 60, 80 nm) with 1 μM of 4-biphenylthiol (BPT). Spectra of BPT, CB[7], and PEG are their corresponding Raman signals from solid material.

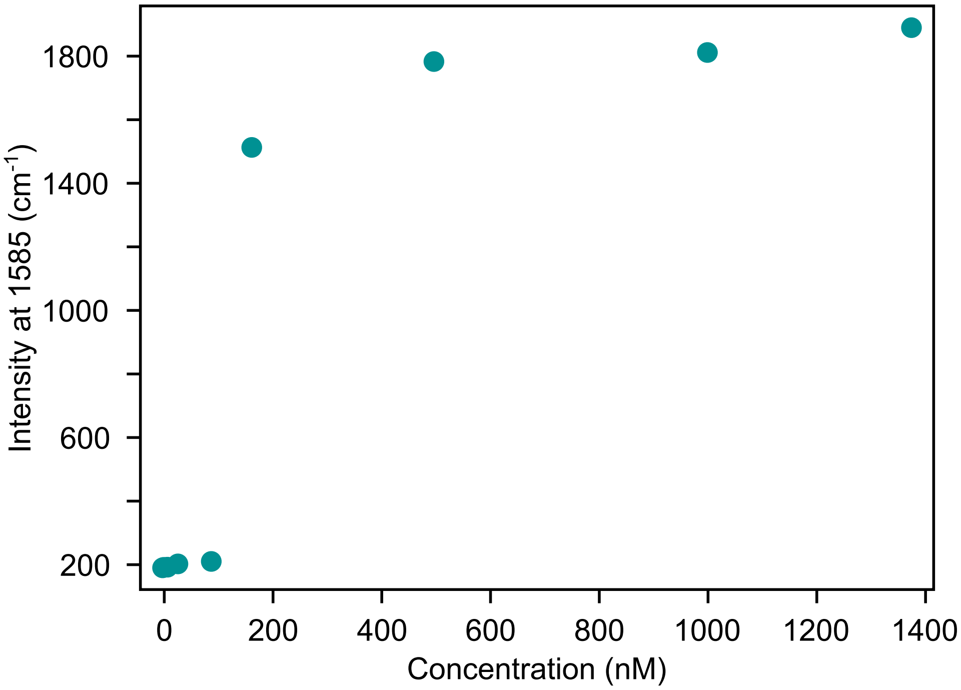

Figure S12: Detection range of 4-biphenylthiol (BPT) (1, 3, 10, 29, 90, 164, 497, 997, 1370 nM) using $AuNP_{80}$:CB[7]:PEG_{6k}.

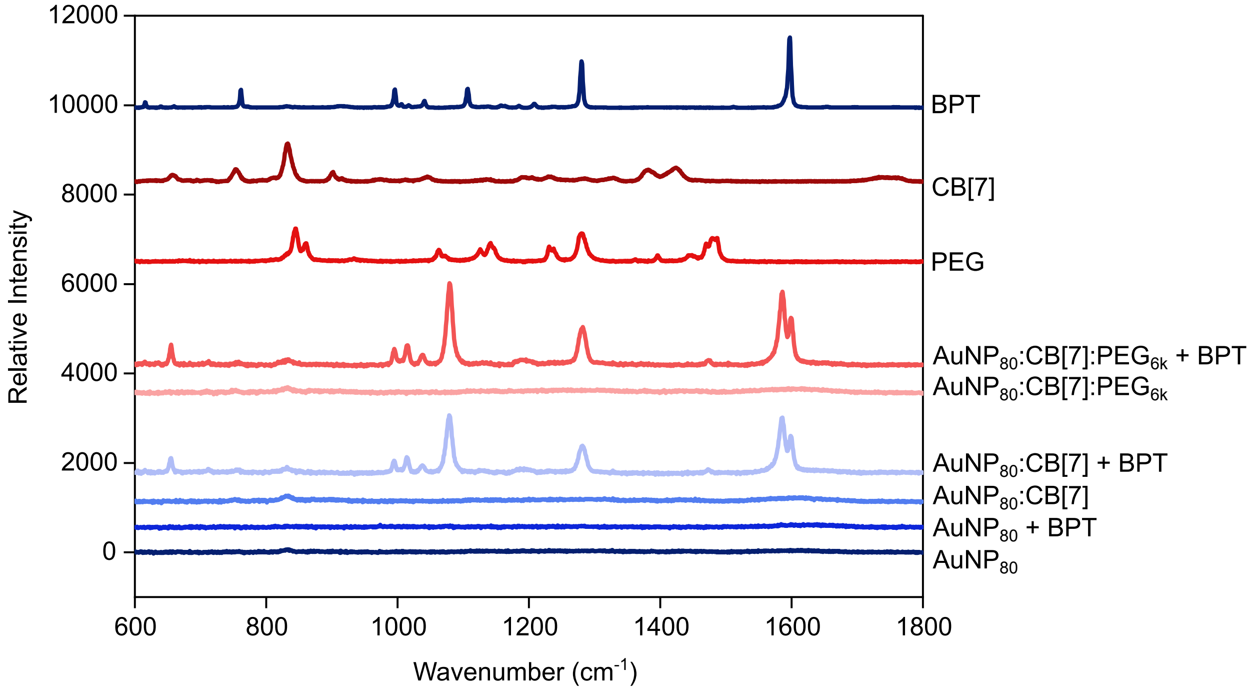

Figure S13: SERS spectra of $AuNP_{80}$, $AuNP_{80}$ + 1 μM 4-biphenylthiol (BPT), $AuNP_{80}$:CB[7], $AuNP_{80}$:CB[7] + 1 μM BPT, $AuNP_{80}$:CB[7]:PEG_{6k}, and $AuNP_{80}$:CB[7]:PEG_{6k} + 1 μM BPT. Spectra of BPT, CB[7], and PEG are their corresponding Raman signals from solid material.

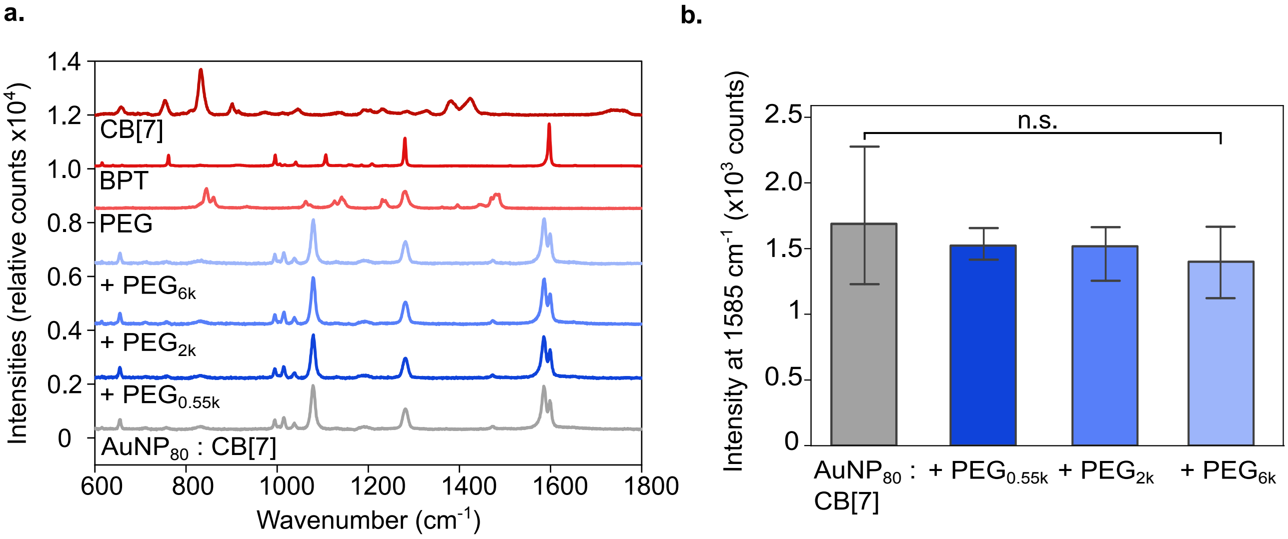

Figure S14: a. SERS spectrum of $AuNP_{80}$:CB[7]:PEG_x (where x = 0.55k, 2k, 6k) with 1 μM of BPT. Spectra of CB[7], PEG (6 kDa), BPT (solid) are their corresponding Raman signals from solid material. **b.** SERS intensities a 1584 cm^{-1} of $AuNP_{80}$:CB[7]:PEG_x (where x = 0.55k, 2k, 6k) with 1 μM of BPT (n = 3)

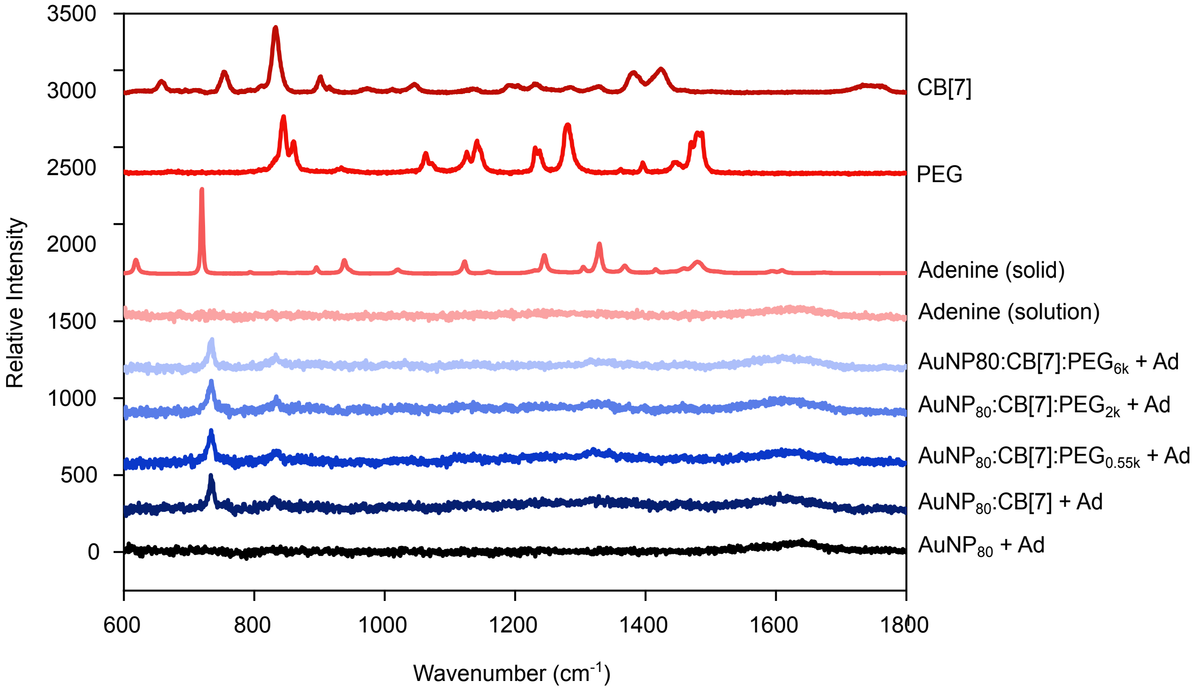

Figure S15: SERS spectrum of $AuNP_{80}$:CB[7]:PEG_x (where x = 0.55k, 2k, 6k), $AuNP_{80}$:CB[7] and $AuNP_{80}$ with 2 mM of Adenine (Ad) . Spectra of CB[7], PEG (6 kDa), Ad (solid) are their corresponding Raman signals from solid material. Adenine (solution) represents the Raman signal of Ad at 2 mM in water.

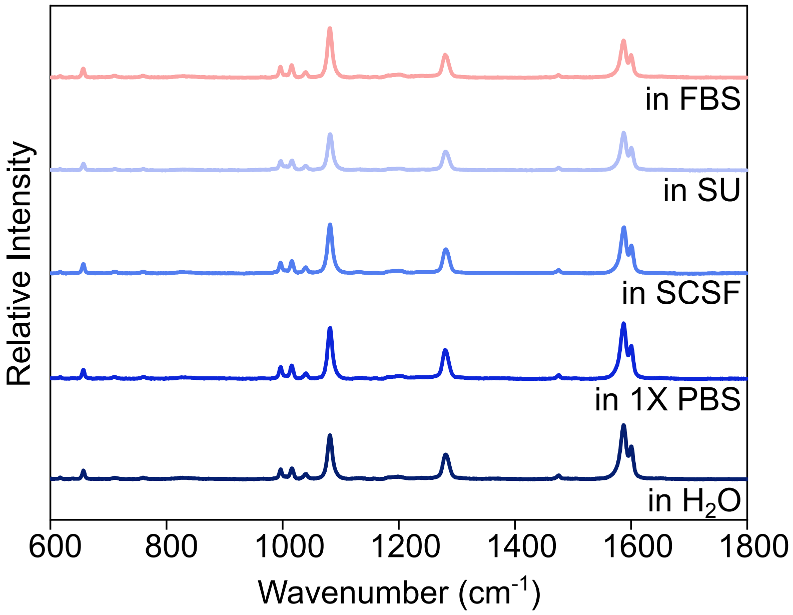

Figure S16: SERS spectrum of $AuNP_{80}$:CB[7]:PEG_x (where x = 0.55k, 2k, 6k) with 1 μM of 4-biphenylthiol (BPT) in H_2O, simulated urine (SU), simulated cerebrospinal fluid (SCSF), 1X phosphate-buffered saline (PBS), and fetal bovine serum (FBS).

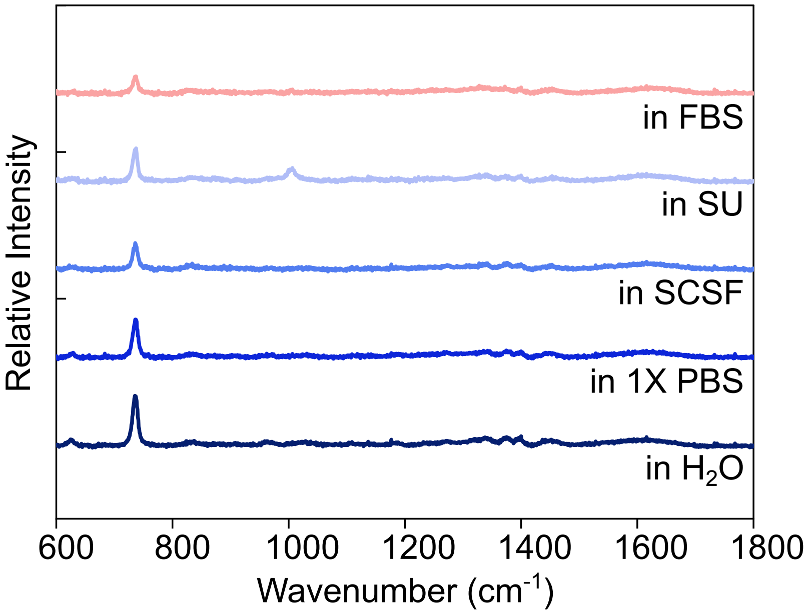

Figure S17: SERS spectrum of $AuNP_{80}$:CB[7]:PEG_x (where x = 0.55k, 2k, 6k) with 2 mM of Adenine (Ad) in H_2O, simulated urine (SU), simulated cerebrospinal fluid (SCSF), 1X phosphate-buffered saline (PBS), and fetal bovine serum (FBS).

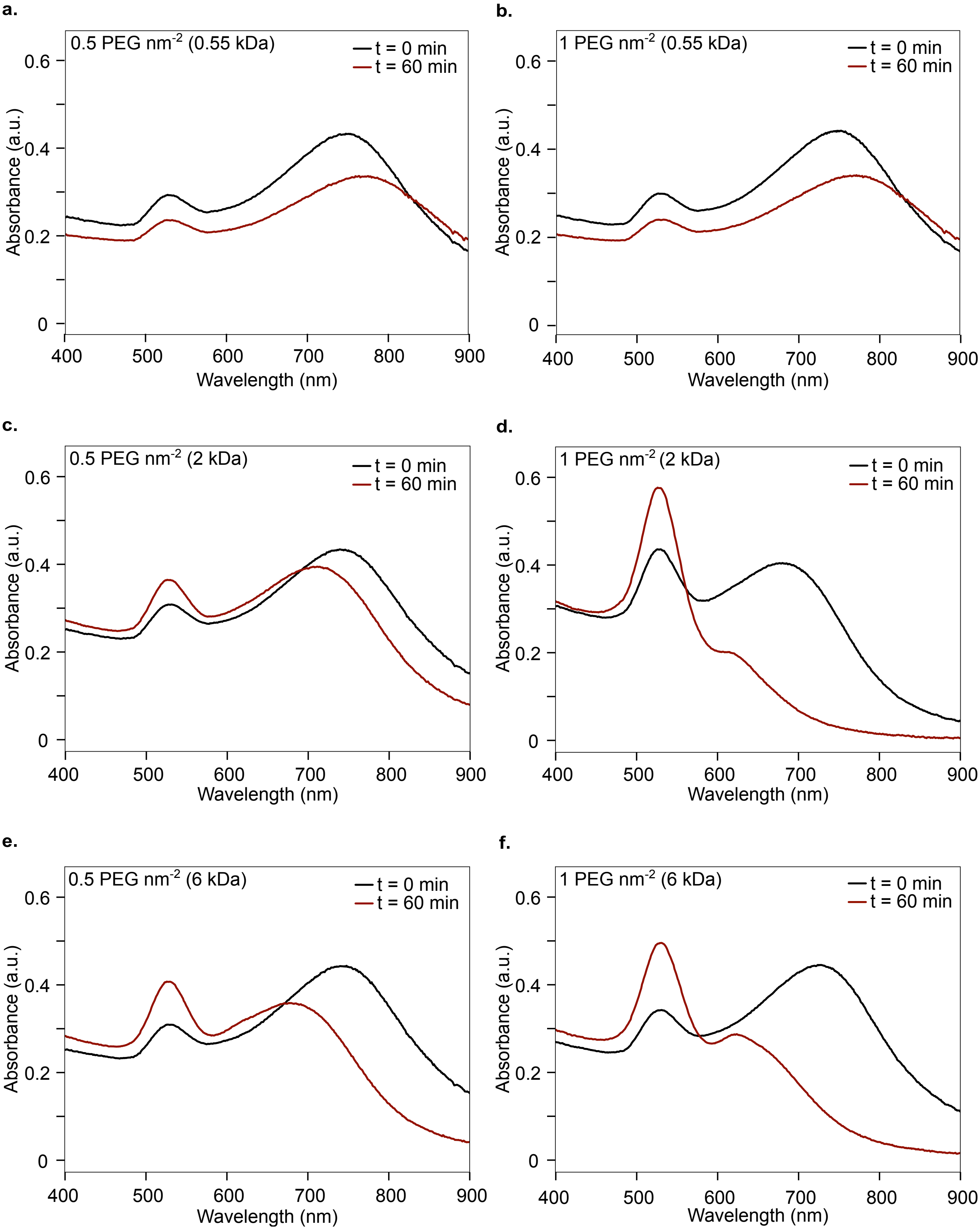

Figure S18: Disassembly of the AuNP$_{40}$:CB[7]:PEG$_{0.55k}$, AuNP$_{40}$:CB[7]:PEG$_{2k}$ and AuNP$_{40}$:CB[7]:PEG$_{6k}$ at grafting densities 1 PEG nm^{-2} (**a., c., and b.**) and 0.5 PEG nm^{-2} (**b., d., and e.**) 60 min after adding the PEG-SH ligand.

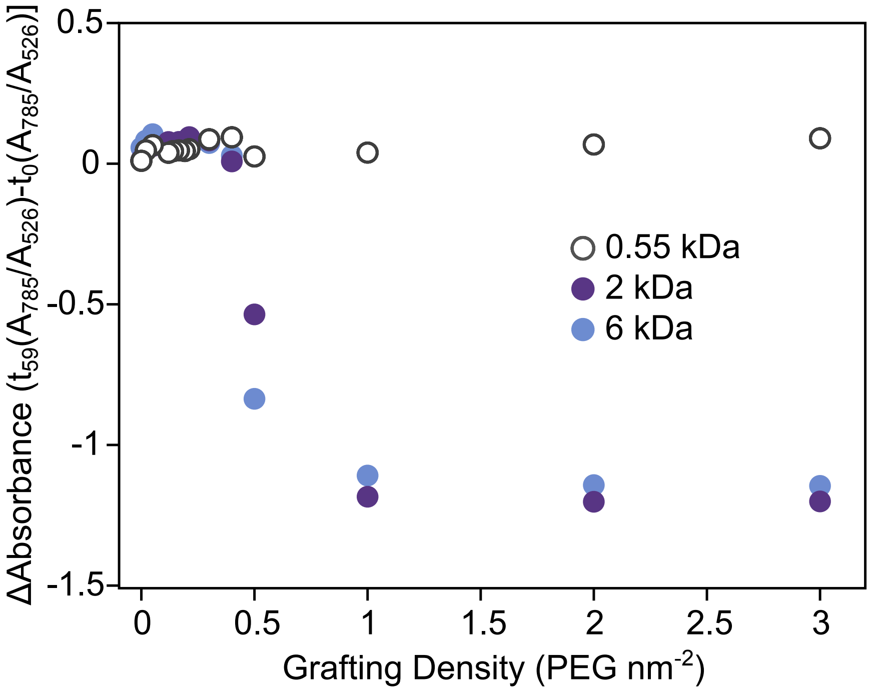

Figure S19: Change in absorbance ratios (A_{785}/A_{526}) at 785 nm and 526 nm from t = 59 to t = 0 based on grafting density of PEG-SH (0.55 kDa, 2 kDa, and 6 kDa; grafting densities from 0 to 3 PEG nm^{-2}) to $AuNP_{40}$:CB[7] aggregates.

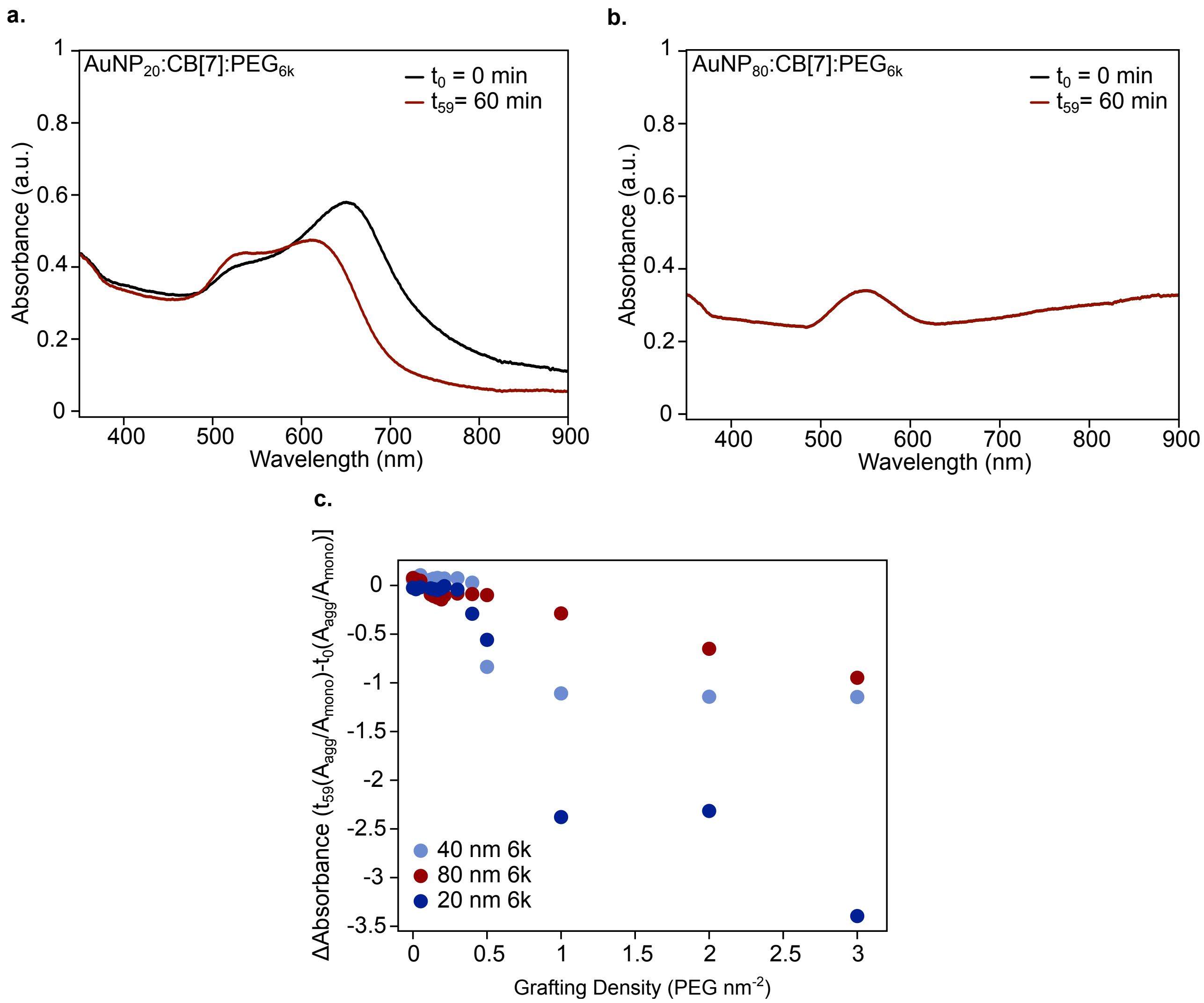

Figure S20: UV-Vis of **a.** AuNP$_{20}$:CB[7]:PEG$_{6k}$ and **b.** AuNP$_{80}$:CB[7]:PEG$_{6k}$ with a grafting density of 0.5 PEG nm^{-2} at t$_0$ = 0 min and t$_{59}$ = 60 min. Change in absorbance ratios (A$_{agg}$/A$_{mono}$) at 854 nm (agg) and 548 nm (mono) for AuNP$_{80}$:CB[7]:PEG$_{6k}$ aggregates, 785 nm (agg) and 526 nm (mono) for AuNP$_{40}$:CB[7]:PEG$_{6k}$ aggregates and 648 nm (agg) and 524 nm (mono) for AuNP$_{20}$:CB[7]:PEG$_{6k}$ aggregates from t = 59 to t = 0 with increasing grafting densities from 0 to 3 PEG nm^{-2}. Data for the 40 nm aggregates from Figure S18.

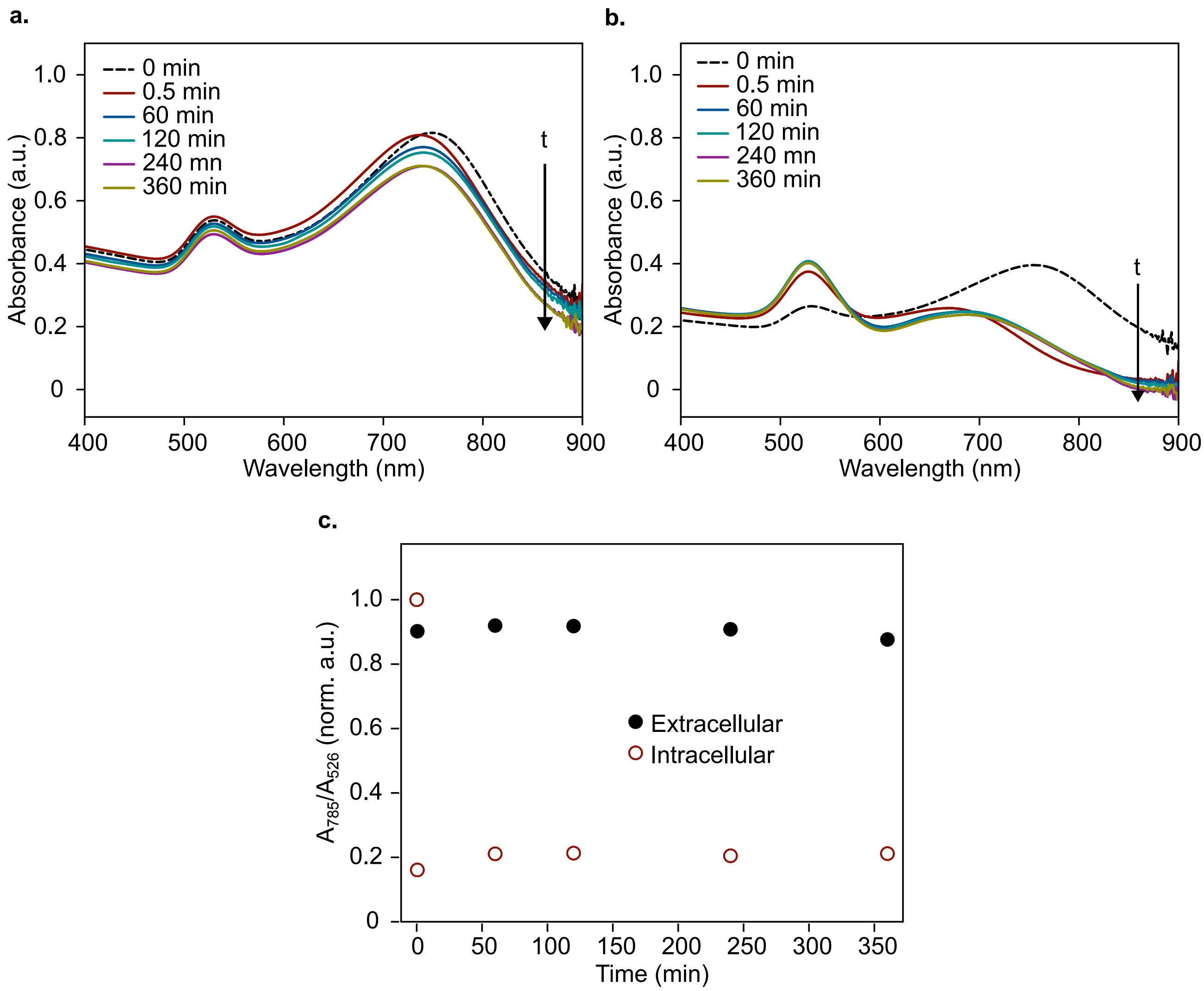

Figure S21: UV-Vis of AuNP$_{40}$:CB[7]:PEG$_{6k}$ in **a.** extracellular, **b.** intracellular concentrations of cysteine (Cys) (5 μM and 200 μM from t = 0 (red line) to t = 6 h (orange line), and **c.** the corresponding absorbance ratio graph of 785 nm to 526 nm over time. Black dashed line represents the control AuNP$_{40}$:CB[7]:PEG$_{6k}$ before exposure to Cys equating to t = 0.

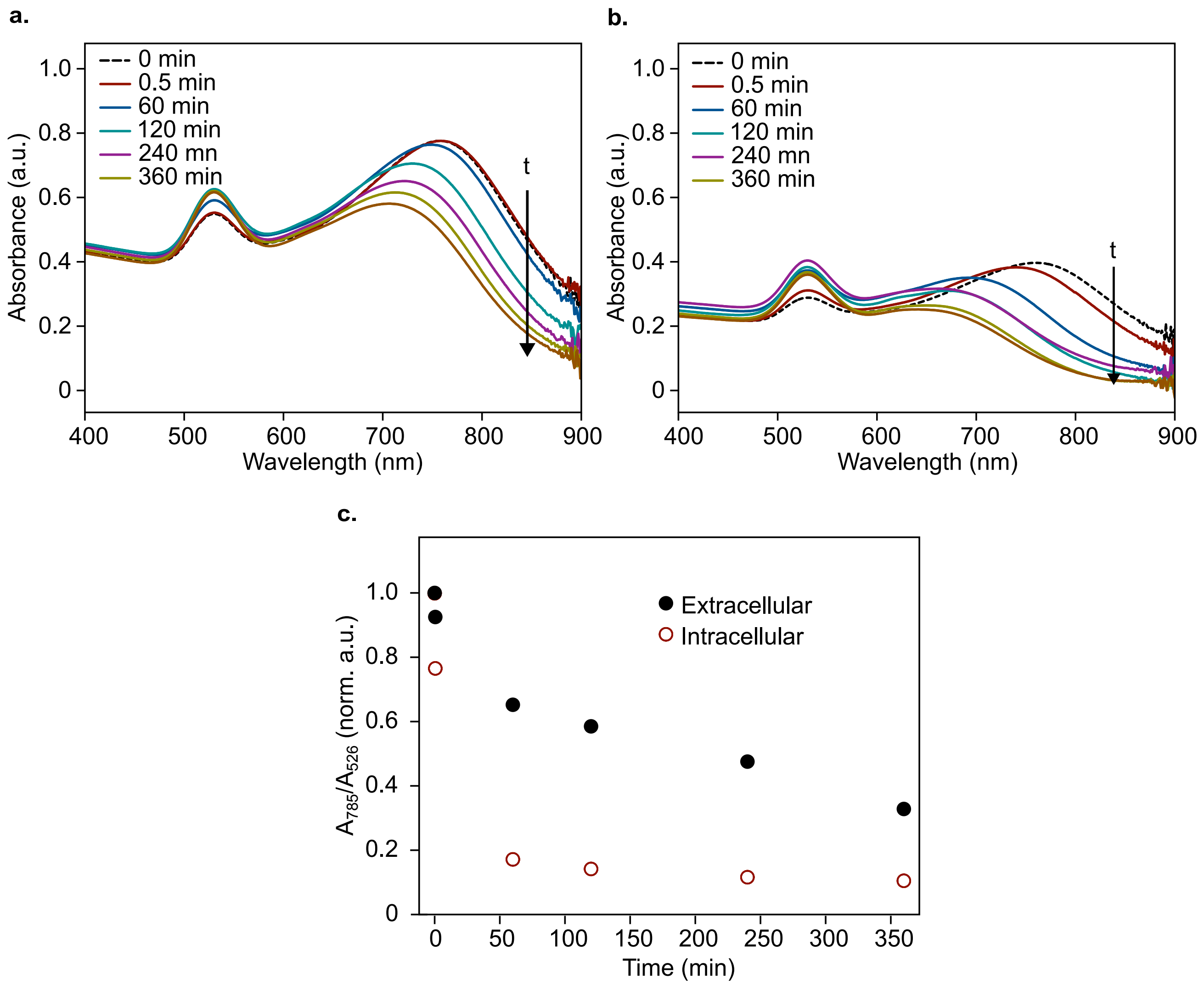

Figure S22: UV-Vis of AuNP$_{40}$:CB[7]:PEG$_{6k}$ in **a.** extracellular, **b.** intracellular concentrations of glutathione (GSH) (3 μM and 1 mM) from t = 0 (red line) to t = 6 h (orange line), and **c.** the corresponding absorbance ratio graph of 785 nm to 526 nm over time. Black dashed line represents the control AuNP$_{40}$:CB[7]:PEG$_{6k}$ before exposure to GSH equating to t = 0.

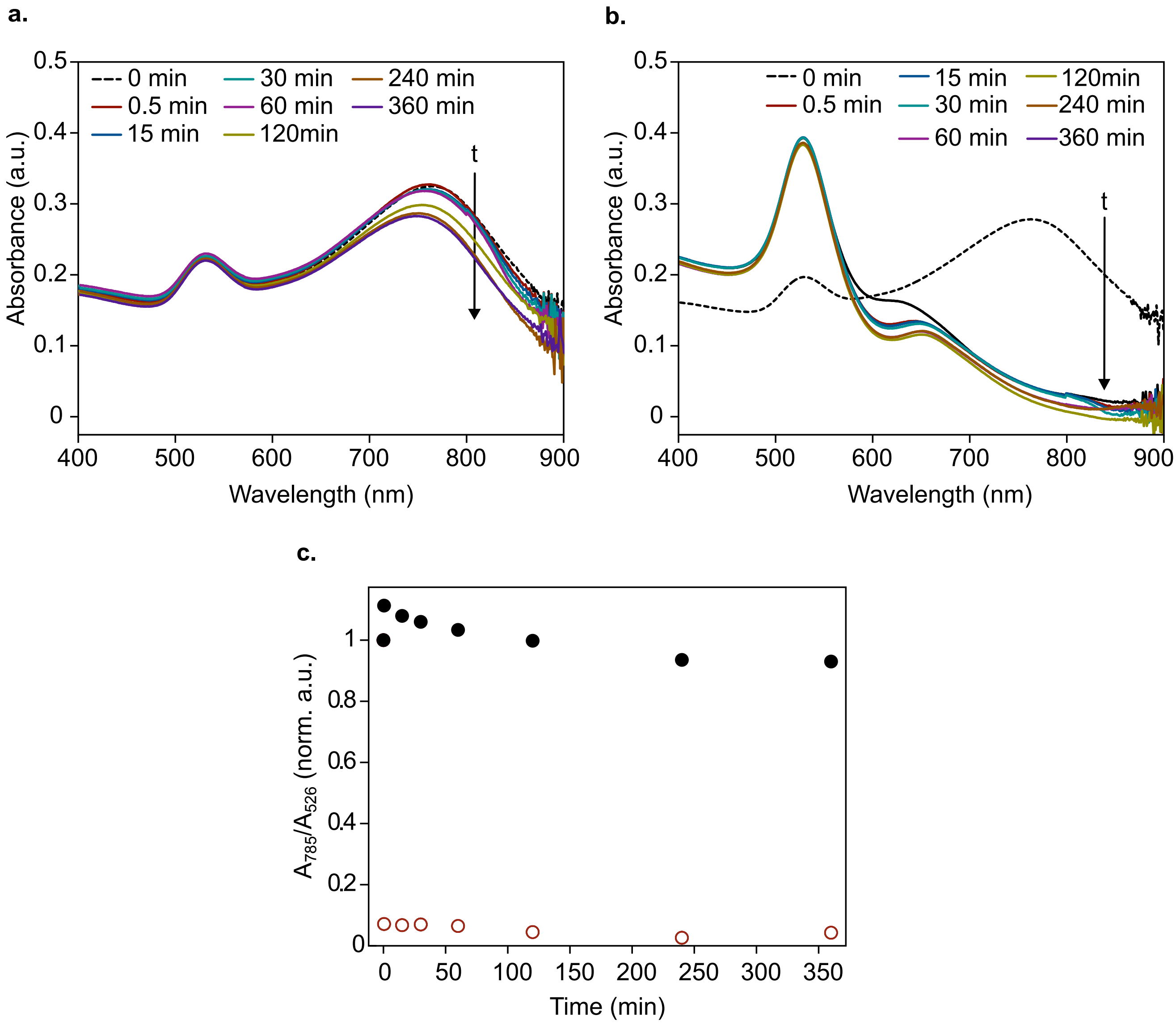

Figure S23: UV-Vis of $AuNP_{40}$:CB[7]:PEG_{6k} stability after the addition of cysteine (Cys) **a.** at 1 μM, **b.** at 500 μM, and the corresponding absorbance ratio graph of 785 nm to 526 nm over time. Arrows show the evolution over time t = 0.5 (red line) to t = 360 min (orange line). Black dashed line represents the control $AuNP_{40}$:CB[7]:PEG_{6k} before exposure to Cys equating to t = 0.

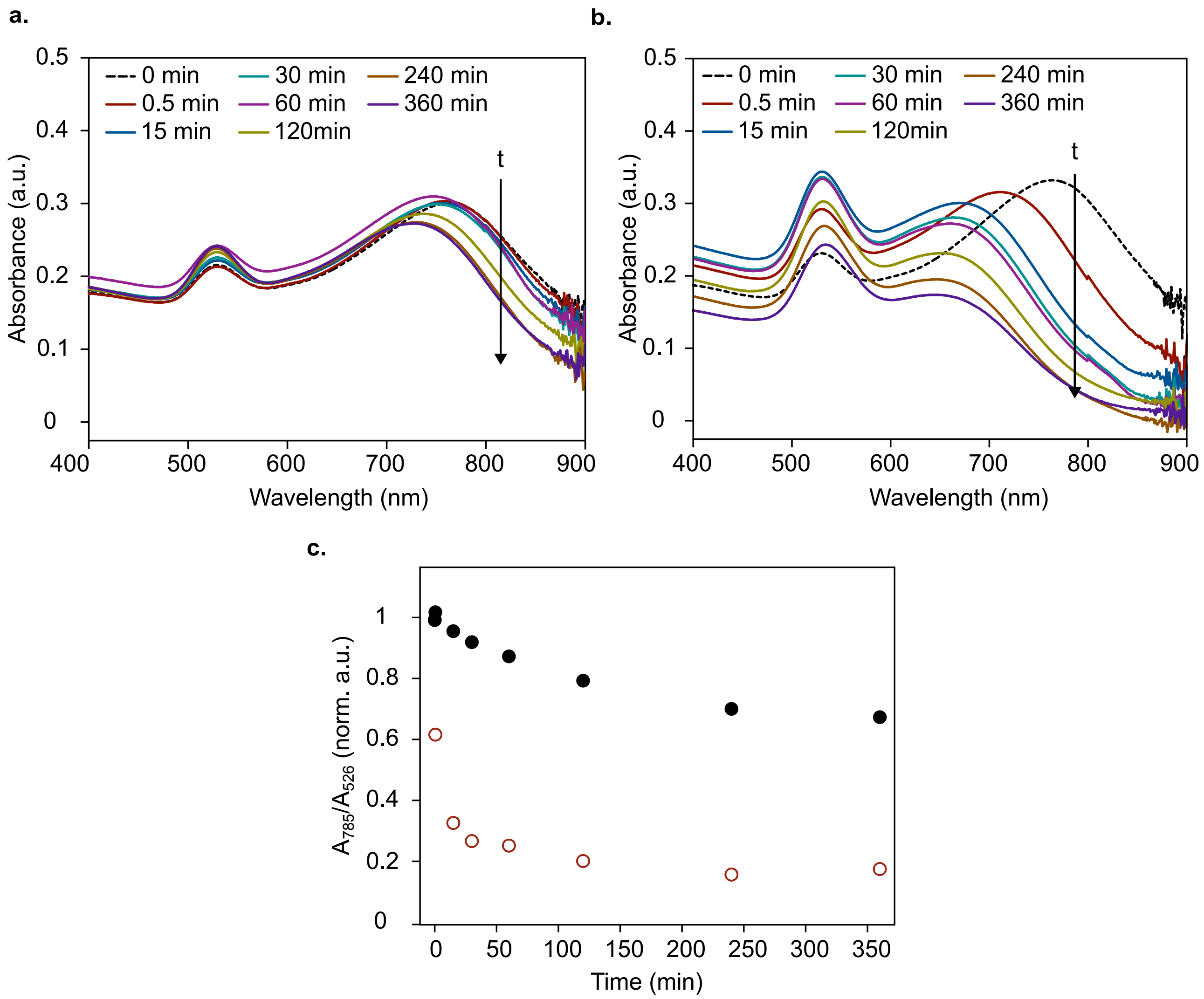

Figure S24: UV-Vis of $AuNP_{40}$:CB[7]:PEG_{6k} stability in glutathione (GSH) **a** at 1 μM, **b.** at 2 mM, and **c.** the corresponding absorbance ratio graph of 785 nm to 526 nm over time. Arrows show the evolution over time t = 0.5 (red line) to t = 360 min (orange line). Black dashed line represents the control $AuNP_{40}$:CB[7]:PEG_{6k} before exposure to GSH equating to t = 0.

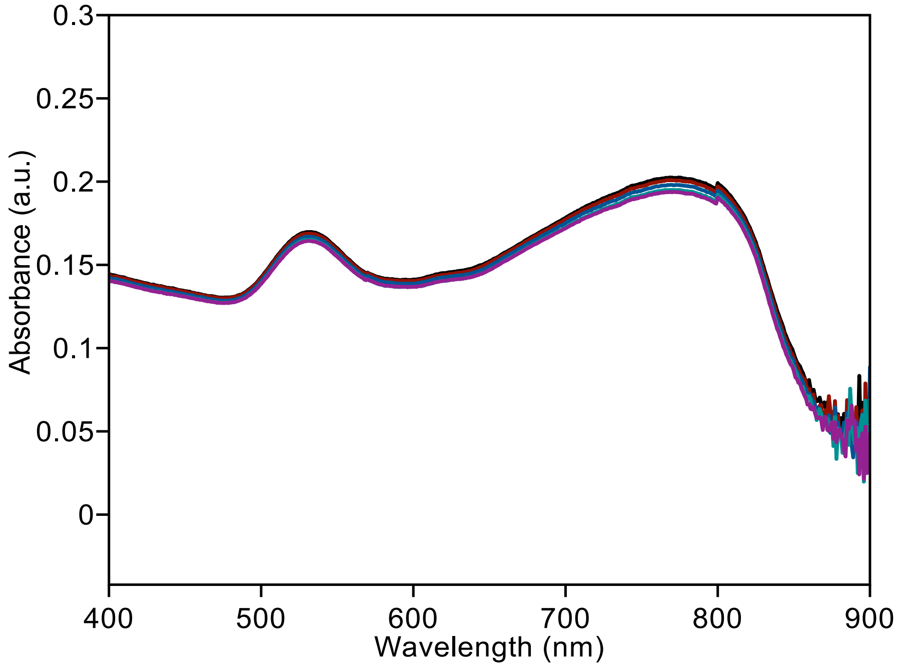

Figure S25: UV-Vis of $AuNP_{40}$:CB[7]:PEG_{6k} in EtOH for 60 min.

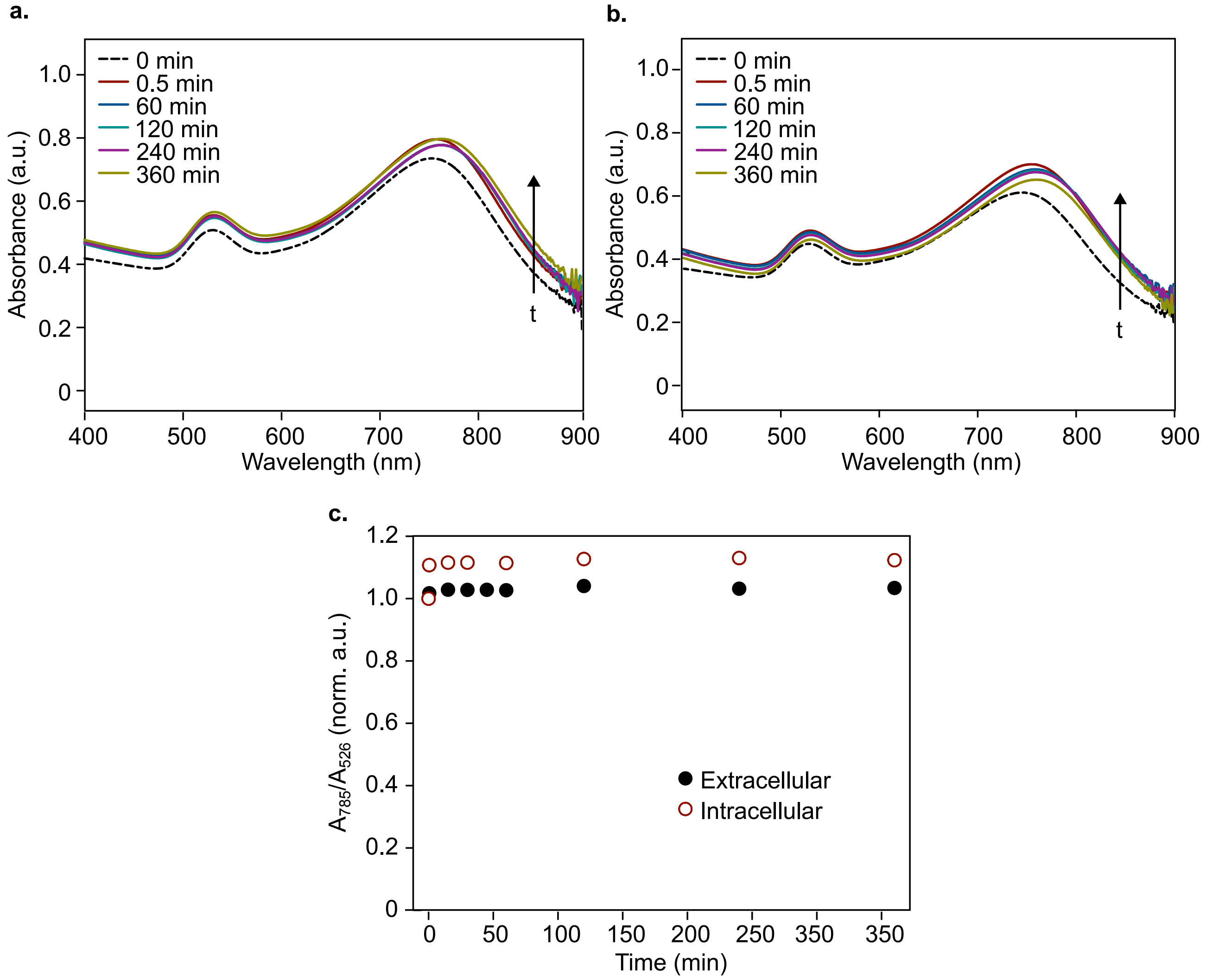

Figure S26: UV-Vis of AuNP$_{40}$:CB[7]:PEG$_{6k}$ at **a.** intracellular, **b.** extracellular concentration of bovine serum albumin (BSA) concentration (200 $mg/\ mL$ and 80 mg/mL), and **b.** the corresponding absorbance ratio graph of 785 nm to 526 nm over time. Black dashed line represents the control AuNP$_{40}$:CB[7]:PEG$_{6k}$ before exposure to BSA equating to t = 0.

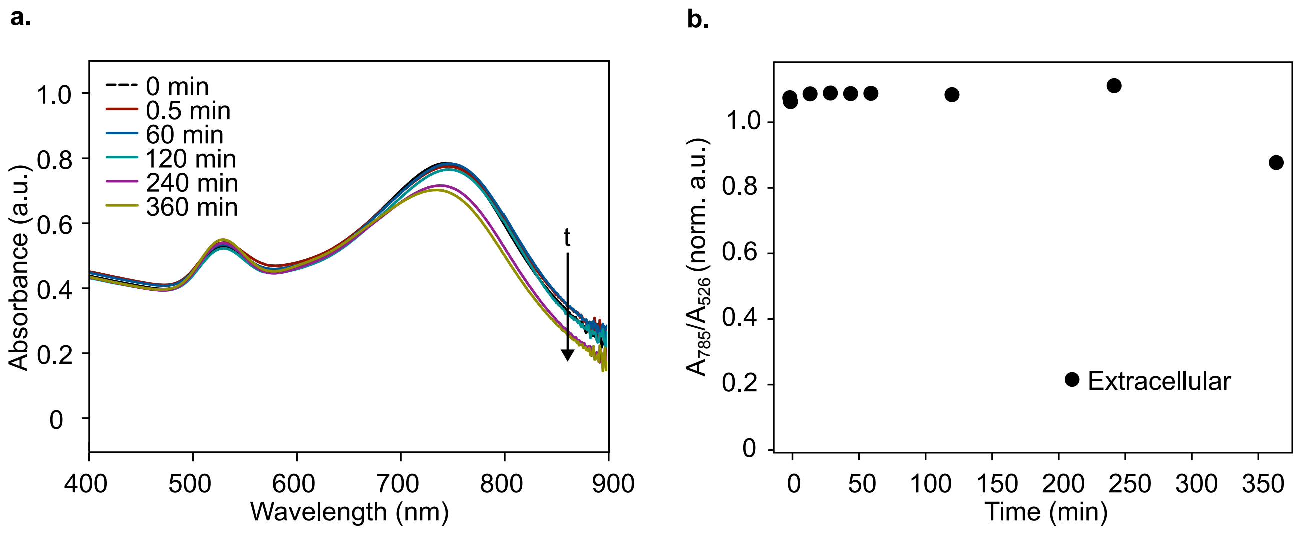

Figure S27: UV-Vis of $AuNP_{40}$:CB[7]:PEG_{6k} at **a.** extracellular concentration of cystine (Cys-Cys) and **b.** the corresponding absorbance ratio graph of 785 nm to 526 nm over time. Black dashed line represents the control $AuNP_{40}$:CB[7]:PEG_{6k} before exposure to Cys-Cys equating to t = 0.

Table S3: Overview of concentration of thiol and non-thiol containing compounds found in plasma and their intra- and extracellular differences.[11–13]

Thiol molecule	Intracellular concentration	Extracellular concentration	Ratio Intra:Extra cellular
Glutathione (GSH)	1,000-10,000 μM	3 μM	300-3,000
Cysteine (Cys)	200 μM	5 μM	40
Cystine (Cys-Cys)	n/a	50 μM	-
Proteins	200 mg/mL	80 mg/mL	2.5

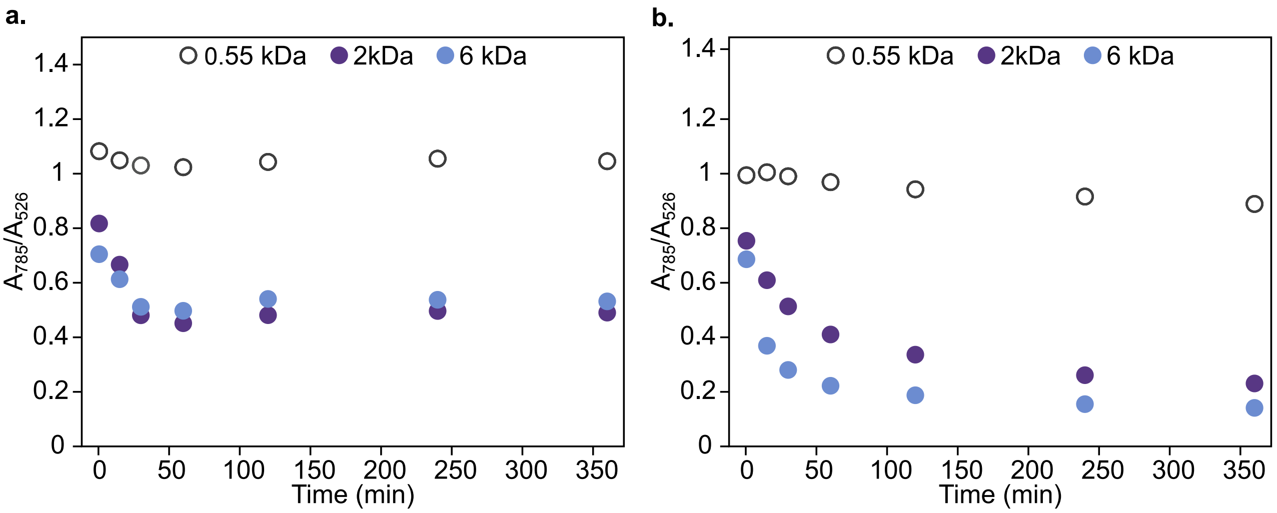

Figure S28: Change in ratio of the absorbance at 785 nm and 526 nm of AuNP$_{40}$:CB[7]:PEG$_x$, where x=0.55k, 2k, and 6k, in **a.** intracellular and **b.** extracellular media.

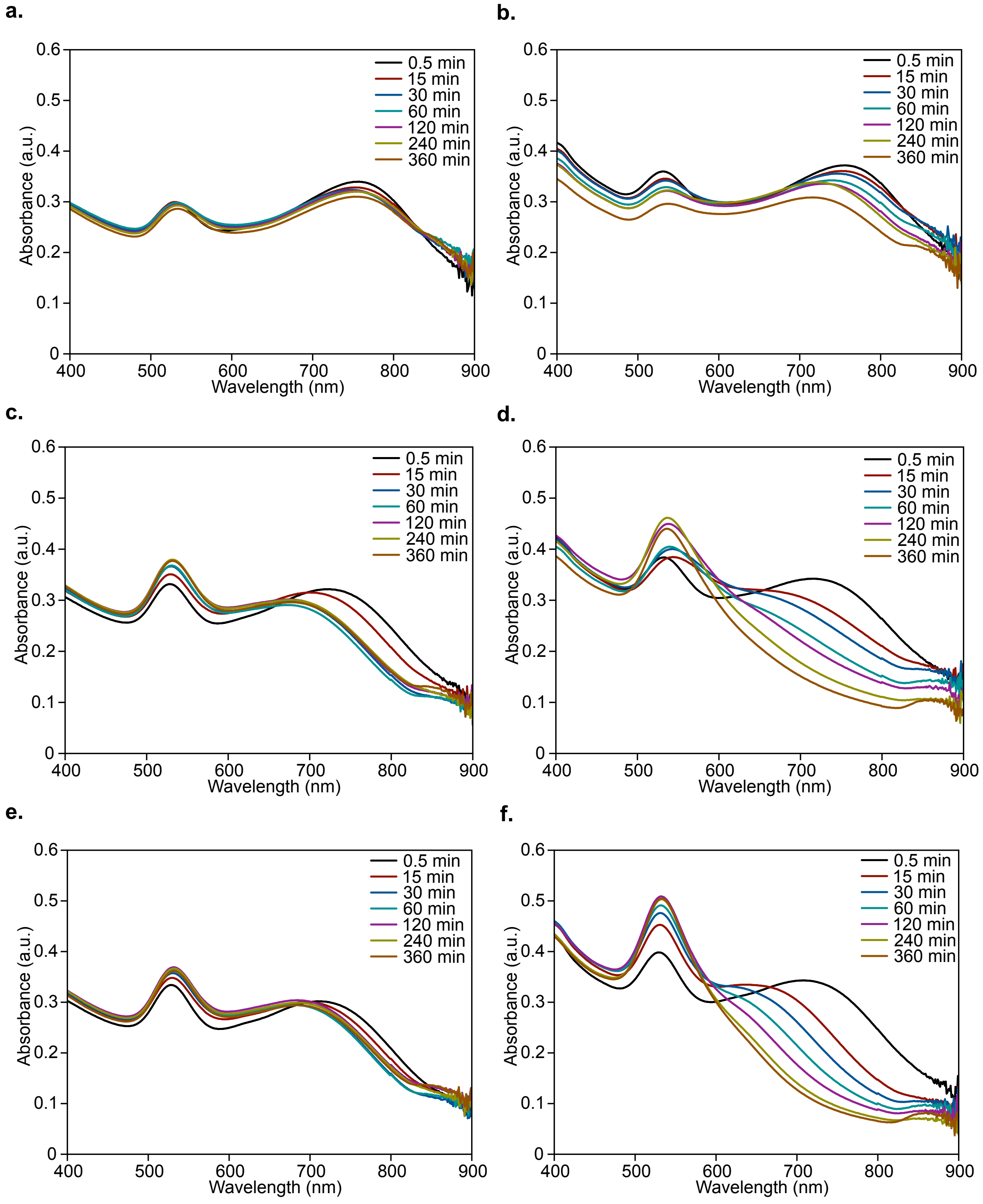

Figure S29: UV-Vis spectra of AuNP$_{40}$:CB[7]:PEG$_{0.55k}$ in **a.** intracellular and **b.** extracellular media, AuNP$_{40}$:CB[7]:PEG$_{2k}$ in **c.** intracellular and **d.** extracellular media, and AuNP$_{40}$:CB[7]:PEG$_{6k}$ in **e.** intracellular and **f.** extracellular media.

References

[1] J. Kim, I.-S. Jung, S.-Y. Kim, E. Lee, J.-K. Kang, S. Sakamoto, K. Yamaguchi, K. Kim, *J. Am. Chem. Soc.* **2000**, *122*, 540.

[2] A. Day, A. P. Arnold, R. J. Blanch, B. Snushall, *J. Org. Chem.* **2001**, *66*, 8094.

[3] I. Badillo-Ramírez, B. Landeros-Rivera, J. M. Saniger, J. Popp, D. Cialla-May, *Analyst* **2023**, *148*, 1848.

[4] J. Turkevich, P. C. Stevenson, J. Hillier, *Discuss. Faraday Soc.* **1951**, *11*, 55.

[5] J. Kimling, M. Maier, B. Okenve, V. Kotaidis, H. Ballot, A. Plech, *J. Phys. Chem. B* **2006**, *110*, 15700, pMID: 16898714.

[6] A. Kenworthy, K. Hristova, D. Needham, T. McIntosh, *Biophys. J.* **1995**, *68*, 1921.

[7] T. Kuhl, D. E. Leckband, D. D. Lasic, J. N. Israelachvili, *Biophys. J.* **1994**, *66*, 1479 .

[8] M. Heuberger, T. Drobek, N. D. Spencer, *Biophys. J.* **2005**, *88*, 495 .

[9] A. K. Dolan, S. F. Edwards, *Proceedings of the Royal Society of London. A. Mathematical and Physical Sciences* **1974**, *337*, 509.

[10] F. Li, F. Pincet, *Langmuir* **2007**, *23*, 12541 .

[11] G. J. McBean, *Antioxidants (Basel)* **2017**, *6*.

[12] W. A. Kleinman, J. P. Richie, *Biochemical Pharmacology* **2000**, *60*, 19.

[13] M. Leeman, J. Choi, S. Hansson, M. U. Storm, L. Nilsson, *Anal. Bioanal. Chem.* **2018**, *410*, 4867 .
